# Supplementary material for: Chronic diseases and multimorbidity among unemployed and employed persons in the Netherlands: a register-based cross-sectional study
Source: BMJ Open. 2020 Jul 2;10(7):e035037. doi: 10.1136/bmjopen-2019-035037 (PMC7333868; doi:10.1136/bmjopen-2019-035037)

Supplementary Table 1. Description of ATC-codes used to identify chronic diseases among unemployed (n=507,583) and employed (n=4,566,644) persons.

Chronic disease	ATC-code	Medication class	Prevalence (%) Unemployed	Prevalence (%) Employed
**Cardiovascular disease**	B01A	Antithrombotic agents	8.5	3.2
	C01	Cardiac agents	1.9	0.7
	C03A	Low-ceiling drugs	3.8	1.5
	C08	Calcium channel blockers	5.4	1.9
	C07	Beta blocking agents	9.6	3.7
	C09A, C09B	ACE inhibitors	7.0	2.8
**Psychological disorders**	N05B	Anxiolytics	6.0	0.9
	N05C	Hypnotics and sedatives	3.4	0.4
	N06A	Antidepressants	14.4	5.0
**Inflammatory and antirheumatic products**	M01A	Anti-inflammatory and antirheumatic products, non-steroids	24.5	15.8
**Respiratory diseases**	R03A, R03C	Adrenergics (inhalants)	10.5	5.8
	R03B, R03D	Other drugs for obstructive airway diseases	4.8	2.3
**Psychotic illness**	N05A	Antipsychotics	6.2	0.8
**Diabetes mellitus**	A10A	Insulins and analogues	2.5	0.8
	A10B	Blood glucose lowering drugs	6.4	1.5

Supplementary Table 2. Combinations and prevalence of multimorbidity of four chronic diseases among unemployed (n=507,583) and employed (n=4,566,644) persons.

**Combination**	**Chronic disease**				**Prevalence**	
	**Cardiovascular diseases (CVD)**	**Psychological disorders (PD)**	**Inflammatory conditions (IC)**	**Respiratory diseases (RD)**	**N (%) Unemployed**	**N (%) Employed**
**1**	1	0	0	0	49,124 (9.7)	248,948 (5.5)
**2**	0	1	0	0	38,549 (7.6)	137,314 (3.0)
**3**	0	0	1	0	64,535 (12.7)	524,452 (11.5)
**4**	0	0	0	1	19,832 (3.9)	181,148 (4.0)
**5**	1	0	0	1	8,611 (1.7)	28,834 (0.6)
**6**	1	0	1	0	15,533 (3.1)	72,591 (1.6)
**7**	1	1	0	0	12,046 (2.4)	25,049 (0.5)
**8**	0	1	1	0	18,098 (3.6)	45,499 (1.0)
**9**	0	0	1	1	9,124 (1.8)	46,741 (1.0)
**10**	0	1	0	1	6,699 (1.3)	14,651 (0.3)
**11**	1	1	1	0	5,995 (1.2)	11,376 (0.2)
**12**	0	1	1	1	4,683 (0.9)	7,041 (0.2)
**13**	1	1	0	1	4,165 (0.8)	4,798 (0.1)
**14**	1	0	1	1	3,722 (0.7)	10,812 (0.2)
**15**	1	1	1	1	2,721 (0.5)	2,792 (0.1)

Supplementary Table 3. The association of employment status with multimorbidity stratified by age.

	**Chronic diseases**		
	**1**	**2**	**≥3**
	**OR (95% CI)**		
Employment status (n=5,074,227)			
Employed	1	1	1
Unemployed	1.30 (1.29-1.31)	1.74 (1.73-1.76)	2.59 (2.56-2.61)
Age 18-30 (n=1,278,018)			
Employed	1	1	1
Unemployed	1.65 (1.61-1.69)	2.84 (2.75-2.93)	5.20 (4.99-5.42)
Age 30-45 (n=1,965,512)			
Employed	1	1	1
Unemployed	1.41 (1.39-1.43)	2.12 (2.09-2.16)	3.52 (3.45-3.59)
Age 45-55 (n=1,180,955)			
Employed	1	1	1
Unemployed	1.22 (1.20-1.23)	1.64 (1.61-1.67)	2.66 (2.62-2.70)
Age 55-65 (n=649,742)			
Employed	1	1	1
Unemployed	1.09 (1.07-1.11)	1.29 (1.26-1.31)	1.78 (1.76-1.81)

Logistic regression analyses were adjusted for sex, educational level and migration background.

Supplementary Figure 1. Prevalence of medicines for psychological disorders (a and b) and cardiovascular diseases (c and d) by age among unemployed (n=507,583) and employed (n=4,566,644) persons in 2016.

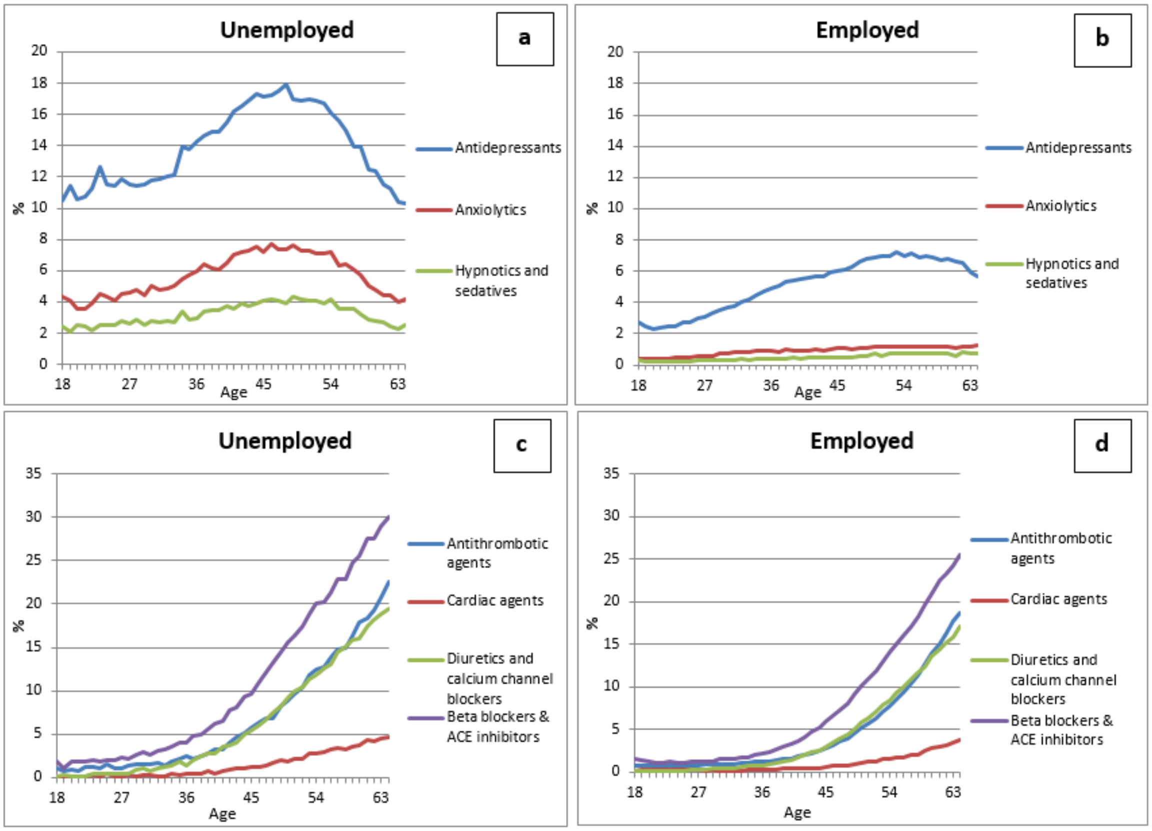

Supplement: Supplementary data [file bmjopen-2019-035037supp001.pdf]
