# Supplementary material for: Chronic diseases and multimorbidity among unemployed and employed persons in the Netherlands: a register-based cross-sectional study
Source: BMJ Open. 2020 Jul 2;10(7):e035037. doi: 10.1136/bmjopen-2019-035037 (PMC7333868; doi:10.1136/bmjopen-2019-035037)
Supplement: Author's manuscript [file bmjopen-2019-035037.draft_revisions.pdf]

BMJ Open is committed to open peer review. As part of this commitment we make the peer review history of every article we publish publicly available.

When an article is published we post the peer reviewers' comments and the authors' responses online. We also post the versions of the paper that were used during peer review. These are the versions that the peer review comments apply to.

The versions of the paper that follow are the versions that were submitted during the peer review process. They are not the versions of record or the final published versions. They should not be cited or distributed as the published version of this manuscript.

BMJ Open is an open access journal and the full, final, typeset and author-corrected version of record of the manuscript is available on our site with no access controls, subscription charges or pay-per-view fees (http://bmjopen.bmj.com).

If you have any questions on BMJ Open's open peer review process please email info.bmjopen@bmj.com

BMJ Open

Multimorbidity of chronic diseases among unemployed and employed persons: a register-based study

Journal:	*BMJ Open*
Manuscript ID	bmjopen-2019-035037
Article Type:	Original research
Date Submitted by the Author:	16-Oct-2019
Complete List of Authors:	Yildiz, Berivan; Erasmus Medical Center, Department of Public Health<br>Schuring, Merel; Erasmus MC, Department of Public Health<br>Knoef, Marike; Leiden University, Department of Economics<br>Burdorf, Alex; Erasmus MC, Department of Public Health
Keywords:	PUBLIC HEALTH, EPIDEMIOLOGY, OCCUPATIONAL & INDUSTRIAL MEDICINE

SCHOLARONE™
Manuscripts

I, the Submitting Author has the right to grant and does grant on behalf of all authors of the Work (as defined in the below author licence), an exclusive licence and/or a non-exclusive licence for contributions from authors who are: i) UK Crown employees; ii) where BMJ has agreed a CC-BY licence shall apply, and/or iii) in accordance with the terms applicable for US Federal Government officers or employees acting as part of their official duties; on a worldwide, perpetual, irrevocable, royalty-free basis to BMJ Publishing Group Ltd ("BMJ") its licensees and where the relevant Journal is co-owned by BMJ to the co-owners of the Journal, to publish the Work in this journal and any other BMJ products and to exploit all rights, as set out in our licence.

The Submitting Author accepts and understands that any supply made under these terms is made by BMJ to the Submitting Author unless you are acting as an employee on behalf of your employer or a postgraduate student of an affiliated institution which is paying any applicable article publishing charge ("APC") for Open Access articles. Where the Submitting Author wishes to make the Work available on an Open Access basis (and intends to pay the relevant APC), the terms of reuse of such Open Access shall be governed by a Creative Commons licence – details of these licences and which Creative Commons licence will apply to this Work are set out in our licence referred to above.

Other than as permitted in any relevant BMJ Author's Self Archiving Policies, I confirm this Work has not been accepted for publication elsewhere, is not being considered for publication elsewhere and does not duplicate material already published. I confirm all authors consent to publication of this Work and authorise the granting of this licence.

For peer review only

Multimorbidity of chronic diseases among unemployed and employed persons: a register-based study

Berivan Yildiz[1], MSc, Merel Schuring[1], PhD, Marike G. Knoef[2], PhD, Alex Burdorf[1], PhD

[1] Department of Public Health, Erasmus University Medical Center, Rotterdam, The Netherlands

[2] Department of Economics, Leiden University, Leiden, The Netherlands

Address of the corresponding author:

M. Schuring, Erasmus University Medical Center, Department of Public Health, P.O. Box 2040, 3000 CA Rotterdam, The Netherlands.

Email: m.schuring@erasmusmc.nl

Phone: 0031 10 703 89 93

Word count: 2946

For peer review only

ABSTRACT

Objectives: The first objective of this study was to describe the prevalence of chronic diseases and multimorbidity among unemployed and employed persons. The second objective was to investigate sociodemographic determinants of chronic diseases and multimorbidity.

Design: Data linkage of cross-sectional nation-wide registries on employment status, medication use and sociodemographic characteristics in 2016 was applied.

Participants: 5,074,227 persons aged 18-65 years were selected with information on employment status, medication use and sociodemographic characteristics.

Outcome measures: Multivariate logistic regression analysis and descriptive statistics were performed to examine associations of employment and sociodemographic characteristics with the prevalence of chronic diseases and multimorbidity. The age-specific prevalence of six common chronic diseases was described, and venn diagrams were applied for multimorbidity among unemployed and employed persons.

Results: Unemployed persons had a higher prevalence of psychological disorders (18.3% vs 5.4%), cardiovascular diseases (20.1% vs 8.9%), inflammatory diseases (24.5% vs 15.8%), and respiratory diseases (11.7% vs 6.5%) than employed persons. Unemployed persons were more likely to have one (OR 1.30 (1.29-1.31)), two (OR 1.74 (1.73-1.76)) and at least three chronic diseases (OR 2.59 (2.56-2.61)) than employed persons. Among unemployed persons, psychological disorders and inflammatory conditions increased with age but declined from middle age onwards, whereas a slight increase was observed among employed persons. Older persons, women, lower educated persons and migrants were more likely to have chronic diseases and multimorbidity.

Conclusion: Large inequalities exist in the prevalence of chronic diseases and multimorbidity among unemployed and employed persons, indicating employment status to be an important determinant of health. Policy measures and health interventions should focus more on promoting employment among unemployed persons with chronic diseases.

For peer review only - http://bmjopen.bmj.com/site/about/guidelines.xhtml

Strengths and limitations of this study

- This is the first study that describes the prevalence of chronic diseases and multimorbidity among unemployed and employed persons, using objective register-based data rather than self-reported health outcomes.
- A strength of this study is applying data linkage of nation-wide registries that capture the whole population, providing enormous statistical power and thus smaller confidence intervals.
- Except for back pain and musculoskeletal disorders, this study investigated a broad range of chronic diseases such as cardiovascular diseases, psychological disorders, diabetes and respiratory diseases.
- Causal effects of having a chronic disease on employment status, or vice versa cannot be distinguished by the use of cross-sectional data.

For peer review only

For peer review only - http://bmjopen.bmj.com/site/about/guidelines.xhtml

INTRODUCTION

The relationship between unemployment and health has been well established (1, 2). In general, unemployed individuals have worse mental and physical health compared to employed individuals (2-5). These health inequalities between employed and unemployed persons can be explained by two different hypotheses. First, according to the causation mechanism, persons who become unemployed will deteriorate in health, whereas unemployed persons who enter paid employment will improve in health (6). Second, the selection mechanism describes that persons who leave paid employment already have lower levels of health before leaving employment compared to those who remain employed, whereas persons who enter paid employment already have a better health status before entering employment compared to persons who remain unemployed (7).

Chronic diseases can affect an individual's employment status due to experienced functional limitations and a poor quality of life (8). Two studies have shown that long term health conditions such as cardiovascular diseases and diabetes were associated with unemployment (9, 10). In addition, a recent systematic review provided evidence that individuals with diabetes were more likely to be out of the labour force (9). However, studies investigating the role of chronic diseases in employment have mostly focused on a single disease, whereas many persons with a chronic disease are likely to suffer from multiple chronic diseases, especially among older age groups. There is increasing evidence that persons with multimorbidity - the co-occurrence of at least two chronic diseases within an individual - may be more likely to have poorer functional outcomes and thus may be also more often out of the labour market than those with a single chronic disease or no chronic disease. For instance, a study among Australian workers with multiple health problems showed that individuals with four or more health problems were far less likely to be employed compared to those with no health condition (11). Another study among persons with back complaints found that the co-occurrence of cardiovascular diseases resulted in a 10-fold increased risk of unemployment compared to those with back complaints alone (12).

So far, findings on the association of chronic diseases and multimorbidity with unemployment have been based mostly on self-reported health outcomes. Self-reported health outcomes are known to be vulnerable to reporting bias and justification bias. Therefore, a more objective approach is preferred in order to make more precise estimations of the prevalence of diseases. One way to objectively

For peer review only - http://bmjopen.bmj.com/site/about/guidelines.xhtml

investigate the presence of chronic diseases is by using pharmacy data (13). Pharmacy data provide a reliable information source and often cover a large population (14). Administrative databases such as drug prescription can be used to identify persons with chronic diseases. So far, only a few studies have used register-based data in order to investigate chronic diseases among unemployed persons. A Danish register-based study found a higher prevalence of mental disorders and cardiovascular disorders among unemployed persons receiving social benefits compared to employed persons (15). In line with this, another register-based study showed that long-term unemployment was associated with a higher risk of antidepressant use (16).

To our knowledge, none of the evidence on the association between multimorbidity and employment status has been based on register-based data. Therefore, the present study aimed to investigate the prevalence of chronic diseases and multimorbidity among unemployed and employed persons. The second aim was to examine associations of employment and sociodemographic characteristics with chronic diseases and multimorbidity. Nation-wide data from the Netherlands on drug prescription, employment status and sociodemographic characteristics in 2016 were used.

For peer review only

For peer review only - http://bmjopen.bmj.com/site/about/guidelines.xhtml

METHODS

Study population and design

Register data covering information on all Dutch residents in 2016 were used. Statistics Netherlands provided individual-level databases on demographics, education, labor market status and prescribed medication. All Dutch residents were pseudonymized using a personal unique number. Data registries were linked at the individual level using these pseudonymized numbers.

Employment status

Individuals aged between 18 and 64 years with available information on employment status were selected (n=10,514,271). This selection captured individuals who were not eligible for exit from paid employment through statutory national retirement schemes. The database on social economic category per month (SECM) provided information on employment status of participants for each month in the year 2016. The main source of income for each consecutive month was used to classify persons as employed or unemployed. Individuals who were in paid employment or self-employed for at least 9 months in 2016 were classified as employed (n=4,566,644). Individuals who were out of the labour market and received either social benefits or unemployment benefits for at least 9 months were classified as unemployed (n=507,583). Individuals who did not meet one of these criteria or had missing data on educational level were excluded (n=5,440,044). In total, 5,074,227 subjects were included in the present study.

Chronic diseases and multimorbidity

The database on medication use in 2016 (Medicijntab) provides information on purchased drugs that were reimbursed by the health care insurances. The drugs were identified using the World Health Organization Anatomical Therapeutic Chemical (ATC) classification codes (17). In line with the study of Huber et al, specific chronic diseases were identified based on these ATC-codes (13). For instance, psychological disorders were identified by ATC-codes that were assigned to drugs such as antidepressants and anxiolytics, whereas inflammatory conditions were identified by the ATC-code that was assigned to non-steroidal anti-inflammatory drugs (NSAID’s) (Supplementary Table 1). In total, 21 chronic diseases were identified. The presence of a chronic disease was dichotomized into having or not having a chronic condition. The total number of chronic diseases was computed for each participant. This measure of multimorbidity of chronic diseases was categorized into four groups: no chronic disease,

For peer review only - http://bmjopen.bmj.com/site/about/guidelines.xhtml

one chronic disease, two chronic diseases, and at least three chronic diseases. Chronic diseases with a prevalence higher than 5% were presented. The total number of chronic diseases included also those with a prevalence lower than 5%, capturing all 21 chronic diseases. All 21 chronic diseases with identifying medications are described by Huber et al (13).

Sociodemographic variables

The databases on sociodemographic characteristics provide information on age, gender, education and ethnicity. A dichotomous variable was computed for employment status (employed vs. unemployed). Educational level was categorized into three educational groups: high (higher vocational training or university), intermediate (higher secondary and intermediate vocational training) and low education (pre-primary education, primary education, and lower secondary education). Age was categorized into four age groups (18-30, 30-45, 45-55, 55-65). Ethnicity was categorized as native Dutch, Moroccan, Turkish, Surinamese and Antillean, other Western, other non-Western.

Analyses

Descriptive statistics were used to describe the prevalence of chronic diseases and multimorbidity among employed and unemployed persons. The association of sociodemographic characteristics (age, sex, education, and ethnic background) and employment status with (multiple) chronic diseases was examined using multivariate logistic regression analysis in the total study population (employed and unemployed persons). Separate logistic regression analyses were done for each dependent variable: i) one chronic disease ii) two chronic diseases and iii) three or more chronic diseases. The association between employment status and multimorbidity stratified by age and educational level was also investigated. To test for possible selection bias, sensitivity analyses were performed by including individuals with missing data on educational level.

The prevalence of multimorbidity was described as the proportion of individuals with all potential combinations of four chronic diseases: cardiovascular diseases, psychological disorders, inflammatory conditions and respiratory diseases. All combinations of co-occurrence between these four chronic diseases were presented in a venn diagram for employed and unemployed persons. The age-specific prevalence of these four chronic diseases among unemployed and employed persons was presented. For cardiovascular diseases and psychological disorders, the age-specific prevalence of medicines used was also presented.

For peer review only - http://bmjopen.bmj.com/site/about/guidelines.xhtml

RESULTS

Unemployed persons were more often older than 45 years (58.2%), female (54.5%), lower educated (51.8%) and from non-Dutch origin (48.1%) compared to employed persons (48.1%). Differences in the prevalence of chronic diseases between unemployed and employed persons were highest for psychological disorders. Compared to employed persons, unemployed persons had a higher prevalence of psychological disorders (18.3% vs 5.4%), cardiovascular diseases (20.1% vs 8.9%), inflammatory conditions (24.5% vs 15.8%), psychotic illness (6.2% vs 0.8%), respiratory diseases (11.7% vs 6.5%) and diabetes (7.2% vs 2.0%). (Table 1)

Table 1. Characteristics of the study population by employment status.

	**Unemployed (n=507.583)**	**Employed (n=4.566.644)**
Age	n (%)	n (%)
18-30	54.807 (10.8)	1.223.211 (26.8)
30-45	157.238 (31.0)	1.808.274 (39.6)
45-55	147.865 (29.1)	1.033.090 (22.6)
55-65	147.673 (29.1)	502.069 (11.0)
Sex		
Male	230.856 (45.4)	2.405.740 (52.7)
Female	276.727 (54.5)	2.160.904 (47.3)
Educational level		
High	73.893 (14.6)	2.022.717 (44.3)
Middle	170.857 (33.7)	1.906.830 (41.8)
Low	262.833 (51.8)	637.097 (14.0)
Ethnicity		
Native Dutch	263.196 (51.9)	3.680.071 (80.6)
Moroccan	35.441 (7.0)	76.841 (1.7)
Turkish	27.131 (5.3)	97.716 (2.1)
Surinamese & Antillean	40.446 (8.0)	145.478 (2.3)
Other Western	37.420 (7.4)	311.005 (6.8)
Other non-Western	103.949 (20.5)	255.533 (5.6)
Chronic conditions		
Inflammatory conditions	124.411 (24.5)	721.304 (15.8)
Cardiovascular diseases	101.917 (20.1)	405.200 (8.9)
Psychological disorders	92.956 (18.3)	248.520 (5.4)
Respiratory diseases	59.557 (11.7)	296.817 (6.5)
Diabetes	36.662 (7.2)	89.382 (2.0)
Psychotic illness	31.308 (6.2)	34.377 (0.8)

For peer review only - http://bmjopen.bmj.com/site/about/guidelines.xhtml

The prevalence of multimorbidity was higher for unemployed persons compared to employed persons. The co-occurrence of both psychological disorders and inflammatory conditions was higher among unemployed persons (6.2%) than among employed persons (1.5%). In addition, the co-occurrence of cardiovascular diseases and inflammatory conditions was higher among unemployed persons (5.7%) compared to employed persons (2.1%). The prevalence of having both cardiovascular diseases and psychological disorders was 4.9% among unemployed persons compared to 0.9% among employed persons. (Figure 1, Supplementary Table 2)

At all ages, unemployed individuals had a higher prevalence of all four chronic diseases compared to employed individuals. The prevalence of psychological disorders increased with age followed by a decrease from middle age onwards among unemployed persons, whereas a slight increase was observed among employed persons. The same pattern was observed for inflammatory conditions. The prevalence of cardiovascular diseases and inflammatory conditions increased with age among both unemployed and employed persons. (Figure 2)

Among unemployed persons, the use of antidepressants, anxiolytics and, hypnotics and sedatives increased with age and decreased from middle age onwards. The prevalence of antidepressants was higher than the use of anxiolytics, hypnotics and sedatives among unemployed persons for all age groups. The use of antithrombotic and cardiac agents, beta blockers and angiotensin-convertin-enzyme (ACE) inhibitors, and diuretics and calcium-channel blockers increased with age among unemployed and employed persons. The prevalence of beta blockers and ACE inhibitors, and antithrombotic agents was higher than diuretics and calcium-channel blockers, and cardiac agents among unemployed persons of all age groups. (Supplementary Figure 1)

Unemployed persons were more likely to have (multiple) chronic conditions compared to employed persons. Unemployed persons were more likely to have one (OR 1.30, 95% CI 1.29-1.31), two (OR 1.74, 95% CI 1.73-1.76) and at least three chronic conditions (OR 2.59, 95% CI 2.56-2.61) than employed persons. Among unemployed persons, 23% had at least three chronic diseases compared to 6% among employed persons. Women (OR 1.49, 95% CI 1.49-1.50), older individuals (OR 1.32 - 2.27), middle and low educated persons (OR 1.32 – 1.49), and non-western migrants (OR 1.01 - 1.34) were also more likely to have a chronic disease. In addition, women (OR 1.61, 95% CI 1.60-1.63), older persons (OR 2.72 - 17.08), lower educated persons (OR 2.11 - 3.32) and non-western migrants (OR 1.02 - 2.03) were also

For peer review only - http://bmjopen.bmj.com/site/about/guidelines.xhtml

more likely to have multiple (≥3) chronic conditions (Table 2). Comparable results were found in sensitivity analyses on the total study population with inclusion of individuals who had missing data (n=7,576,196) on educational level.

Within all age groups, unemployed persons had a higher risk of having one, two and at least three chronic diseases than employed persons. Especially, among persons aged 18-30 years, unemployed persons were more likely to have one (OR 1.65, 95% CI 1.61-1.69), two (OR 2.84 (95% CI 2.75-2.93) and at least three (OR 5.20, 95% CI 4.99-5.42) chronic diseases compared to employed persons. These effect estimates were lower among older age groups. (Supplementary Table 3)

For peer review only

For peer review only - http://bmjopen.bmj.com/site/about/guidelines.xhtml

Table 2. The association of sociodemographic characteristics with multimorbidity among the total population (n=5.074.227).

	**Number of chronic conditions**					
	**1**	**OR (95% CI)**	**2**	**OR (95% CI)**	**≥3**	**OR (95% CI)**
Employment status						
Employed (n=4.566.644)	1.007.275 (22.1%)	1	394.396 (8.6%)	1	287.660 (6.3%)	1
Unemployed (n=507.583)	118.688 (23.4%)	1.30 (1.29-1.31)	79.719 (15.7%)	1.74 (1.73-1.76)	115.764 (22.8%)	2.59 (2.56-2.61)
Gender						
Male (n=2.636.596)	524.931 (19.9%)	1	214.039 (8.1%)	1	179.540 (6.8%)	1
Female (n=2.437.631)	601.032 (24.7%)	1.49 (1.49 - 1.50)	260.076 (10.7%)	1.60 (1.59-1.61)	223.884 (9.2%)	1.61 (1.60-1.63)
Age						
18-30 (n=1.278.018)	249.361 (19.5%)	1	66.065 (5.2%)	1	25.443 (2.0%)	1
30-45 (n=1.965.512)	435.651 (22.2%)	1.32 (1.31-1.33)	154.922 (7.9%)	1.78 (1.77-1.80)	92.888 (4.7%)	2.72 (2.68-2.76)
45-55 (n=1.180.955)	286.880 (24.3%)	1.80 (1.79-1.81)	146.145 (12.4%)	3.38 (3.35-3.41)	143.542 (12.2%)	8.00 (7.88-8.11)
55-65 (n=649.742)	154.071 (23.7%)	2.27 (2.26-2.29)	106.983 (16.5%)	5.62 (5.55-5.68)	141.551 (21.8%)	17.08 (16.83-17.33)
Educational level						
High (n=2.096.610)	436.035 (20.8%)	1	150.868 (7.2%)	1	89.730 (4.3%)	1
Middle (n=2.077.687)	478.089 (23.0%)	1.32 (1.31-1.32)	202.938 (9.8%)	1.62 (1.60-1.63)	162.416 (7.8%)	2.11 (2.09-2.13)
Low (n=899.930)	211.839 (23.5%)	1.49 (1.48-1.50)	120.309 (13.4%)	2.06 (2.04-2.07)	151.278 (16.8%)	3.32 (3.29-3.36)
Ethnicity						
Dutch (n=3.943.267)	869.203 (22.0%)	1	356.489 (9.0%)	1	286.169 (7.3%)	1
Moroccan (n=112.282)	27.621 (24.6%)	1.26 (1.25-1.28)	13.278 (11.8%)	1.38 (1.35-1.41)	13.840 (12.3%)	1.49 (1.45-1.52)
Turkish (n=124.847)	30.208 (24.2%)	1.34 (1.32-1.36)	14.818 (11.9%)	1.58 (1.55-1.61)	17.208 (13.8%)	2.03 (1.99-2.07)
Surinamese & Antillean (n=185.924)	42.837 (23.0%)	1.10 (1.09-1.11)	20.541 (11.0%)	1.19 (1.17-1.21)	22.149 (11.9%)	1.39 (1.37-1.41)
Other Western (n=348.425)	73.959 (21.2%)	0.91 (0.90-0.91)	30.235 (8.7%)	0.88 (0.87-0.89)	25.345 (7.3%)	0.87 (0.85-0.88)
Other Non-Western (n=359.482)	82.135 (22.8%)	1.01 (1.00-1.02)	38.754 (10.8%)	1.01 (0.99-1.02)	38.713 (10.8%)	1.02 (1.01-1.03)

For peer review only - http://bmjopen.bmj.com/site/about/guidelines.xhtml

DISCUSSION

In this large register-based study, unemployed persons had a higher prevalence of cardiovascular diseases, psychological disorders, inflammatory conditions, respiratory diseases and multimorbidity compared to employed persons. Between unemployed and employed persons, the largest differences were observed for cardiovascular diseases and psychological disorders. The prevalence of psychological disorders and inflammatory conditions increased with age but declined from middle age onwards among unemployed persons. Women, older individuals, lower educated persons and non-western migrants were more likely to have one chronic disease as well as multiple chronic diseases. Among younger persons (18-30 years), a stronger association between chronic disease and unemployment was found compared to higher age groups.

The higher prevalence of chronic diseases among unemployed persons in the current study is in line with other studies showing a negative association between unemployment and health. Several studies have shown that unemployed persons have a poorer mental and physical health status (2, 5, 18). For instance, unemployed persons had high risks of common mental disorders such as depression (19-21). Our study added to the current literature by comparing the age-specific prevalence of chronic diseases between unemployed and employed persons. A remarkable finding was the different pattern of the age-specific prevalence of psychological disorders between unemployed and employed persons. Among both employed and unemployed persons, the prevalence of psychological disorders increased with age. However, this increase was more profound among unemployed persons with a peak around middle age. This pattern among unemployed persons can be explained by studies arguing that before and after middle age, individuals tend to suffer less from unemployment compared to persons of middle age (22). Persons of middle age often have family responsibilities, increasing the financial importance of a job, whereas younger and older persons experience less financial pressure and thus less psychological distress due to unemployment (23). The other way around, it can also be that the combination of family responsibilities and work lead to pressure and cause both health problems as well as unemployment. Furthermore, it has also been hypothesized that persons of middle age are more likely to aim for a successful career which leads to employment being more important for their mental health than it is for older persons who are almost finishing their careers, and for younger persons who have recently entered paid employment (24). The age-specific prevalence of antidepressants use, anxiolytics, and hypnotics and sedatives among unemployed persons confirms this theory by showing the highest

For peer review only - http://bmjopen.bmj.com/site/about/guidelines.xhtml

prevalence at middle age and a lower prevalence at younger and older ages. It was checked whether the selection of unemployed individuals could explain the decline in the prevalence of antidepressants use, anxiolytics, hypnotics and sedatives after the age of 50. This does not appear to be the case as also among other groups, such as disabled individuals, a decline was observed.

Another interesting finding was an increase in the prevalence of inflammatory conditions with increasing age followed by a decrease from middle age onwards among unemployed persons. This finding can be explained by the medicines NSAID's that have been used to identify inflammatory conditions as a chronic disease in the present study. NSAID's are pain killers with anti-inflammatory effects and are known to cause serious adverse effects (25). In the Netherlands, NSAID's are therefore cautiously or not prescribed among individuals aged 60 years or older, who suffer from cardiovascular diseases and already use other medicines for other (chronic) health conditions. In the present study, the prevalence of cardiovascular diseases and multimorbidity was higher among unemployed persons compared to employed persons. Thus, it may be that NSAID's are less prescribed among older persons because of multimorbidity with CVD and associated polypharmacy – the use of multiple medicines. Therefore, the higher prevalence of multimorbidity among unemployed persons may explain the decrease in the prevalence of inflammatory conditions with increasing age.

The strength of the present study is the use of register-based data, which is a more objective method to investigate the association between health and unemployment. In earlier studies, the relationship between health and unemployment has often been examined using self-reported outcomes of health and disability (18). However, a major concern of self-reported health outcomes is that unemployed individuals may over-report their level of disability or work limitations to justify that they are not in paid employment (26). In the current study, this problem has been minimized using register-based data on medication use that has been collected independently of the study. Another strength of the current study is that in our register-based data the whole Dutch population is involved and therefore the data provide enormous statistical power resulting in smaller confidence intervals. Lastly, the use of register-based data is less expensive since no additional efforts have to be made for data collection and no concerns are present about health-related non-response.

Register-based data also have some limitations. Although a broad range of chronic diseases has been investigated in this study, several conditions that are associated with unemployment have not been

For peer review only - http://bmjopen.bmj.com/site/about/guidelines.xhtml

included, such as back pain or musculoskeletal disorders. In the current study, it was not possible to identify these chronic conditions by the use of medication data. For instance, medication that is prescribed for back pain includes over the counter pain killers such as paracetamol or ibuprofen. However, since pain killers are used for various forms of bodily pain and no information was available regarding the reason of prescription, it was not possible to identify these health conditions. Yet, conditions such as back pain and musculoskeletal disorders are known to lead to exit from paid employment (12) and therefore should be investigated among unemployed persons in future studies. Another limitation of this study was that almost 33% of the eligible study population was excluded due to missing data on educational level. However, sensitivity analyses including individuals with missing data showed comparable results.

This study showed that health inequalities exist between unemployed and employed persons. Specifically, among the younger age group, a strong association of chronic diseases and multimorbidity with unemployment was found. Due to the cross-sectional design, it was not possible to investigate causal relationships between unemployment and health. However, several studies have shown beneficial effects of employment on health (27, 28). Although it may be a challenge to increase employment rates among unemployed persons with chronic diseases, it may lead to improvements in health. In order to reduce health inequalities between unemployed and employed persons, it is therefore important that re-integration policies focus more on promoting employment among unemployed persons with chronic diseases.

In conclusion, the current study showed that unemployed persons more often have chronic diseases and multimorbidity than employed persons, indicating employment status to be an important determinant of health. Policy measures are needed to improve health and promote employment among unemployed persons.

For peer review only

Acknowledgements

-

Contributors

BY and MS prepared the data. BY performed the statistical analysis, drafted and revised the article. MS, MGL and AB participated in the analyses. MS, MGK and AB critically reviewed the manuscript. All authors approved the final version.

Funding

This study was funded by ZonMw (The Netherlands Organisation for Health Research and Development).

Competing interests

The authors declare no conflict of interest.

Patient consent for publication

Not required.

Data sharing statement

No data is available.

For peer review only

References

1. Janlert U. Unemployment as a disease and diseases of the unemployed. Scand J Work Environ Health. 1997;23 Suppl 3:79-83.
2. McKee-Ryan F, Song Z, Wanberg CR, Kinicki AJ. Psychological and physical well-being during unemployment: a meta-analytic study. J Appl Psychol. 2005;90(1):53-76.
3. Bartley M, Sacker A, Clarke P. Employment status, employment conditions, and limiting illness: prospective evidence from the British household panel survey 1991-2001. J Epidemiol Community Health. 2004;58(6):501-6.
4. Wanberg CR. The individual experience of unemployment. Annu Rev Psychol. 2012;63:369-96.
5. Roos E, Lahelma E, Saastamoinen P, Elstad JI. The association of employment status and family status with health among women and men in four Nordic countries. Scand J Public Health. 2005;33(4):250-60.
6. Schuring M, Robroek SJ, Lingsma HF, Burdorf A. Educational differences in trajectories of self-rated health before, during, and after entering or leaving paid employment in the European workforce. Scand J Work Environ Health. 2015;41(5):441-50.
7. Thomas C, Benzeval M, Stansfeld SA. Employment transitions and mental health: an analysis from the British household panel survey. J Epidemiol Community Health. 2005;59(3):243-9.
8. Varekamp I, van Dijk FJH, Kroll LE. Workers with a chronic disease and work disability. Bundesgesundheitsblatt - Gesundheitsforschung - Gesundheitsschutz. 2013;56(3):406-14.
9. Pedron S, Emmert-Fees K, Laxy M, Schwettmann L. The impact of diabetes on labour market participation: a systematic review of results and methods. BMC public health. 2019;19(1):25-.
10. Leal J, Luengo-Fernandez R, Gray A, Petersen S, Rayner M. Economic burden of cardiovascular diseases in the enlarged European Union. Eur Heart J. 2006;27(13):1610-9.
11. Schofield DJ, Callander EJ, Shrestha RN, Passey ME, Percival R, Kelly SJ. Multiple chronic health conditions and their link with labour force participation and economic status. PLoS One. 2013;8(11):e79108.
12. Schofield DJ, Callander EJ, Shrestha RN, Passey ME, Percival R, Kelly SJ. Association between co-morbidities and labour force participation amongst persons with back problems. Pain. 2012;153(10):2068-72.
13. Huber CA, Szucs TD, Rapold R, Reich O. Identifying patients with chronic conditions using pharmacy data in Switzerland: an updated mapping approach to the classification of medications. BMC Public Health. 2013;13:1030.
14. Zhan C, Miller M. Administrative data based patient safety research: a critical review. Quality & safety in health care. 2003;12(Suppl 2):ii58-ii63.
15. Andersen I, Brønnum-Hansen H, Kriegbaum M, Hougaard CØ, Hansen FK, Diderichsen F. Increasing illness among people out of labor market–A Danish register-based study. Social Science & Medicine. 2016;156:21-8.
16. Virtanen M, Kivimaki M, Ferrie JE, Elovainio M, Honkonen T, Pentti J, et al. Temporary employment and antidepressant medication: a register linkage study. J Psychiatr Res. 2008;42(3):221-9.
17. World Health O. WHO Collaborating Centre for Drug Statistics Methodology: ATC classification index with DDDs and Guidelines for ATC classification and DDD assignment. Oslo, Norway: Norwegian Institute of Public Health. 2006.
18. Luo J, Qu Z, Rockett I, Zhang X. Employment status and self-rated health in north-western China. Public Health. 2010;124(3):174-9.

For peer review only - http://bmjopen.bmj.com/site/about/guidelines.xhtml

19. Stankunas M, Kalediene R, Starkuviene S, Kapustinskiene V. Duration of unemployment and depression: a cross-sectional survey in Lithuania. BMC Public Health. 2006;6:174.
20. Khlat M, Sermet C, Le Pape A. Increased prevalence of depression, smoking, heavy drinking and use of psycho-active drugs among unemployed men in France. European journal of epidemiology. 2004;19(5):445-51.
21. Ford E, Clark C, McManus S, Harris J, Jenkins R, Bebbington P, et al. Common mental disorders, unemployment and welfare benefits in England. Public health. 2010;124(12):675-81.
22. Broomhall HS, Winefield AH. A comparison of the affective well-being of young and middle-aged unemployed men matched for length of unemployment. British Journal of Medical Psychology. 1990;63(1):43-52.
23. Jackson PR, Warr PB. Unemployment and psychological ill-health: the moderating role of duration and age. Psychological Medicine. 1984;14(3):605-14.
24. Lahelma E. Unemployment, re-employment and mental well-being. A panel survey of industrial jobseekers in Finland. Scandinavian Journal of Social Medicine, Supplement. 1989;17(43):1-170.
25. Sostres C, Gargallo CJ, Arroyo MT, Lanas A. Adverse effects of non-steroidal anti-inflammatory drugs (NSAIDs, aspirin and coxibs) on upper gastrointestinal tract. Best Practice & Research Clinical Gastroenterology. 2010;24(2):121-32.
26. Bound J. Self-Reported Versus Objective Measures of Health in Retirement Models. The Journal of Human Resources. 1991;26(1):106-38.
27. Schuring M, Robroek SJ, Burdorf A. The benefits of paid employment among persons with common mental health problems: evidence for the selection and causation mechanism. Scand J Work Environ Health. 2017;43(6):540-9.
28. van der Noordt M, H IJ, Droomers M, Proper KI. Health effects of employment: a systematic review of prospective studies. Occup Environ Med. 2014;71(10):730-6.

For peer review only

For peer review only - http://bmjopen.bmj.com/site/about/guidelines.xhtml

Figure 1. Multimorbidity of cardiovascular diseases (CVD), psychological disorders (PD), inflammatory conditions (IC) and respiratory diseases (RD) among unemployed (n=507.583) and employed persons (n=4.566.644).

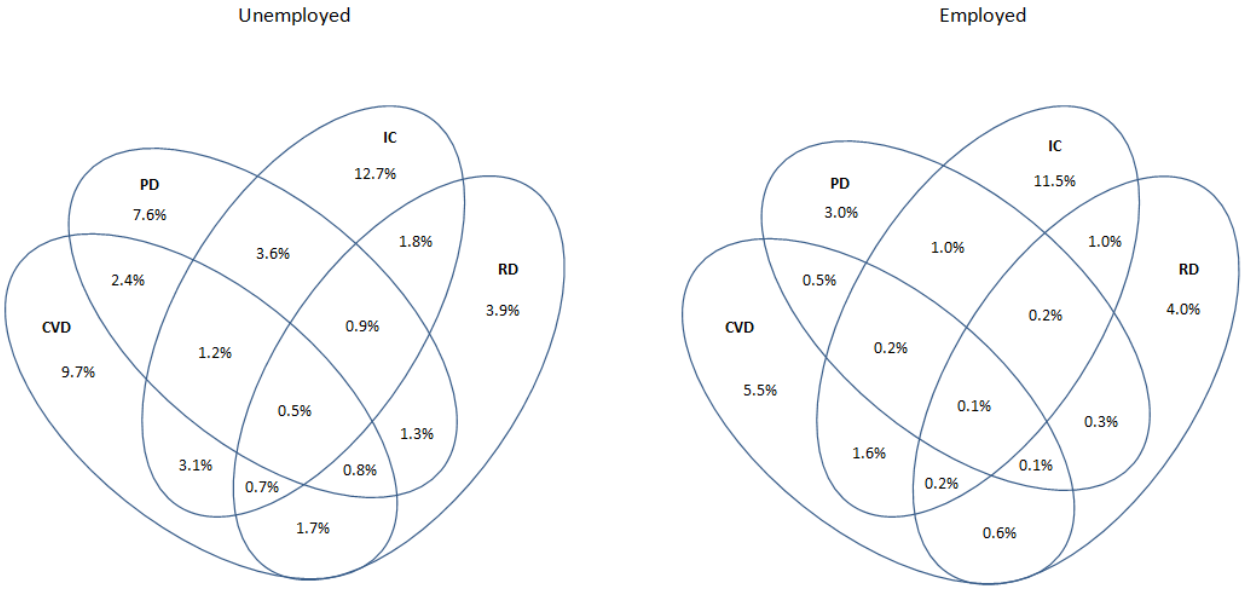

For peer review only

Figure 2. Prevalence of four chronic diseases by age among unemployed (n=507.583) and employed (n=4.566.644) persons in 2016.

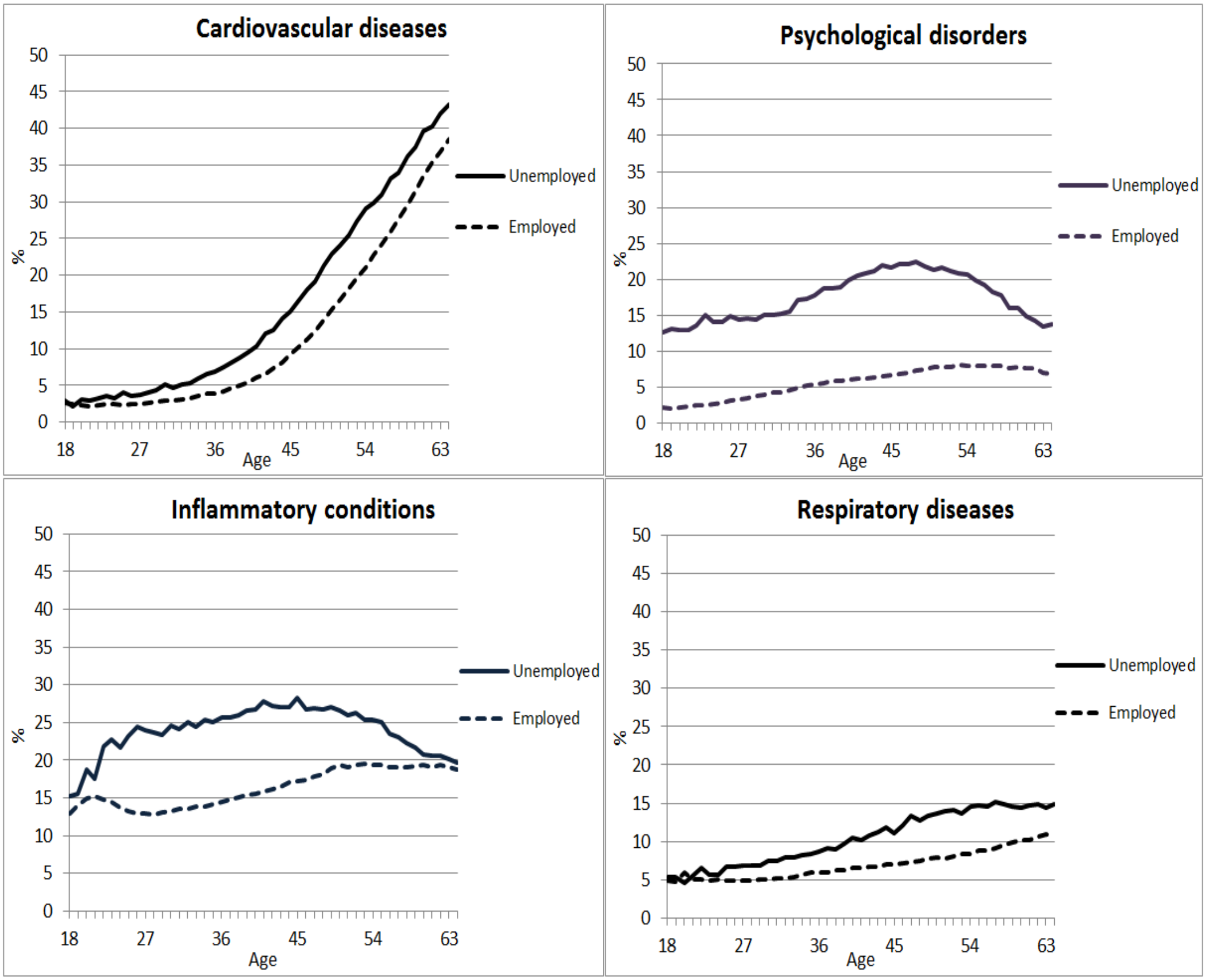

Supplementary Table 1. Description of ATC-codes used to identify chronic diseases among unemployed (n=507.583) and employed (n=4.566.644) persons.

Chronic disease	ATC-code	Medication class	Prevalence (%) Unemployed	Prevalence (%) Employed
**Cardiovascular disease**	B01A	Antithrombotic agents	8.5	3.2
	C01	Cardiac agents	1.9	0.7
	C03A	Low-ceiling drugs	3.8	1.5
	C08	Calcium channel blockers	5.4	1.9
	C07	Beta blocking agents	9.6	3.7
	C09A, C09B	ACE inhibitors	7.0	2.8
**Psychological disorders**	N05B	Anxiolytics	6.0	0.9
	N05C	Hypnotics and sedatives	3.4	0.4
	N06A	Antidepressants	14.4	5.0
**Inflammatory and antirheumatic products**	M01A	Anti-inflammatory and antirheumatic products, non-steroids	24.5	15.8
**Respiratory diseases**	R03A, R03C	Adrenergics (inhalants)	10.5	5.8
	R03B, R03D	Other drugs for obstructive airway diseases	4.8	2.3
**Psychotic illness**	N05A	Antipsychotics	6.2	0.8
**Diabetes mellitus**	A10A	Insulins and analogues	2.5	0.8
	A10B	Blood glucose lowering drugs	6.4	1.5

For peer review only

Supplementary Table 2. Combinations and prevalence of multimorbidity of four chronic diseases among unemployed (n=507.583) and employed (n=4.566.644) persons.

Combination	Chronic disease				Prevalence	
	Cardiovascular diseases (CVD)	Psychological disorders (PD)	Inflammatory conditions (IC)	Respiratory diseases (RD)	N (%) Unemployed	N (%) Employed
**1**	1	0	0	0	49.124 (9.7)	248.948 (5.5)
**2**	0	1	0	0	38.549 (7.6)	137.314 (3.0)
**3**	0	0	1	0	64.535 (12.7)	524.452 (11.5)
**4**	0	0	0	1	19.832 (3.9)	181.148 (4.0)
**5**	1	0	0	1	8.611 (1.7)	28.834 (0.6)
**6**	1	0	1	0	15.533 (3.1)	72.591 (1.6)
**7**	1	1	0	0	12.046 (2.4)	25.049 (0.5)
**8**	0	1	1	0	18.098 (3.6)	45.499 (1.0)
**9**	0	0	1	1	9.124 (1.8)	46.741 (1.0)
**10**	0	1	0	1	6.699 (1.3)	14.651 (0.3)
**11**	1	1	1	0	5.995 (1.2)	11.376 (0.2)
**12**	0	1	1	1	4.683 (0.9)	7.041 (0.2)
**13**	1	1	0	1	4.165 (0.8)	4.798 (0.1)
**14**	1	0	1	1	3.722 (0.7)	10.812 (0.2)
**15**	1	1	1	1	2.721 (0.5)	2.792 (0.1)

For peer review only

Supplementary Table 3. The association of employment status with multimorbidity stratified by age. *

	**Chronic diseases**		
	**1**	**2**	**≥3**
	**OR (95% CI)**		
Employment status (n=5.074.227)			
Employed	1	1	1
Unemployed	1.30 (1.29-1.31)	1.74 (1.73-1.76)	2.59 (2.56-2.61)
Age 18-30 (n=1.278.018)			
Employed	1	1	1
Unemployed	1.65 (1.61-1.69)	2.84 (2.75-2.93)	5.20 (4.99-5.42)
Age 30-45 (n=1.965.512)			
Employed	1	1	1
Unemployed	1.41 (1.39-1.43)	2.12 (2.09-2.16)	3.52 (3.45-3.59)
Age 45-55 (n=1.180.955)			
Employed	1	1	1
Unemployed	1.22 (1.20-1.23)	1.64 (1.61-1.67)	2.66 (2.62-2.70)
Age 55-65 (n=649.742)			
Employed	1	1	1
Unemployed	1.09 (1.07-1.11)	1.29 (1.26-1.31)	1.78 (1.76-1.81)

*Analyses were adjusted for sex, educational level and ethnic background.

For peer review only

Supplementary Figure 1. Prevalence of medicines for psychological disorders (a and b) and cardiovascular diseases (c and d) by age among unemployed (n=507.583) and employed (n=4.566.644) persons in 2016.

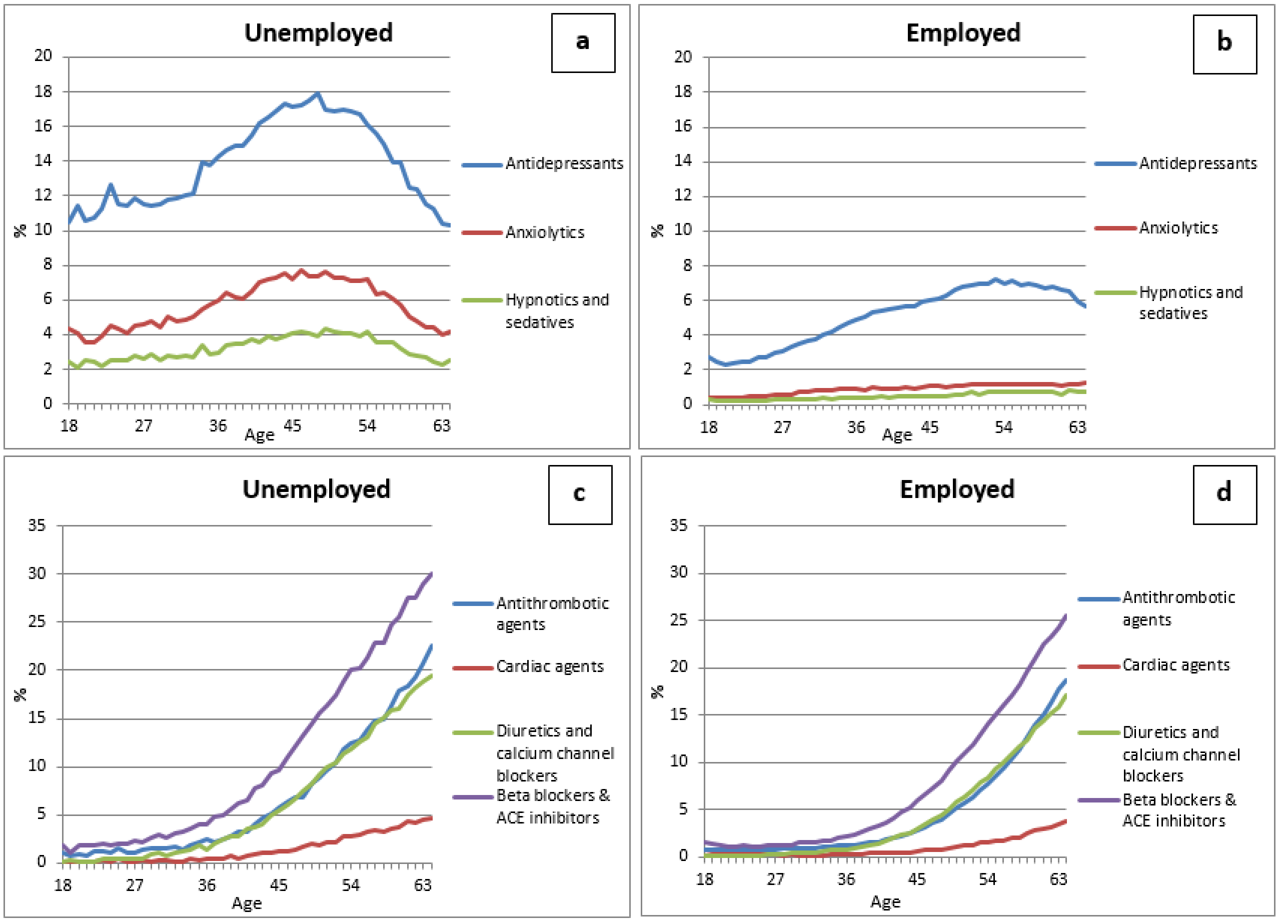

For peer review only - http://bmjopen.bmj.com/site/about/guidelines.xhtml

STROBE Statement—Checklist of items that should be included in reports of ***cross-sectional studies***

	Item No	Recommendation	Page No
**Title and abstract**	1	(*a*) Indicate the study's design with a commonly used term in the title or the abstract	0/1
		(*b*) Provide in the abstract an informative and balanced summary of what was done and what was found	1
**Introduction**			
Background/rationale	2	Explain the scientific background and rationale for the investigation being reported	3
Objectives	3	State specific objectives, including any prespecified hypotheses	4
**Methods**			
Study design	4	Present key elements of study design early in the paper	5
Setting	5	Describe the setting, locations, and relevant dates, including periods of recruitment, exposure, follow-up, and data collection	5
Participants	6	(*a*) Give the eligibility criteria, and the sources and methods of selection of participants	5
Variables	7	Clearly define all outcomes, exposures, predictors, potential confounders, and effect modifiers. Give diagnostic criteria, if applicable	5/6
Data sources/ measurement	8*	For each variable of interest, give sources of data and details of methods of assessment (measurement). Describe comparability of assessment methods if there is more than one group	*6*
Bias	9	Describe any efforts to address potential sources of bias	6
Study size	10	Explain how the study size was arrived at	5
Quantitative variables	11	Explain how quantitative variables were handled in the analyses. If applicable, describe which groupings were chosen and why	6
Statistical methods	12	(*a*) Describe all statistical methods, including those used to control for confounding	6
		(*b*) Describe any methods used to examine subgroups and interactions	6
		(*c*) Explain how missing data were addressed	6
		(*d*) If applicable, describe analytical methods taking account of sampling strategy	n.a
		(*e*) Describe any sensitivity analyses	6
**Results**			
Participants	13*	(a) Report numbers of individuals at each stage of study—eg numbers potentially eligible, examined for eligibility, confirmed eligible, included in the study, completing follow-up, and analysed	n.a/ 7
		(b) Give reasons for non-participation at each stage	6
		(c) Consider use of a flow diagram	
Descriptive data	14*	(a) Give characteristics of study participants (eg demographic, clinical, social) and information on exposures and potential confounders	7
		(b) Indicate number of participants with missing data for each variable of interest	5
Outcome data	15*	Report numbers of outcome events or summary measures	7-9
Main results	16	(*a*) Give unadjusted estimates and, if applicable, confounder-adjusted estimates and their precision (eg, 95% confidence interval). Make clear which confounders were adjusted for and why they were included	8-9

For peer review only - http://bmjopen.bmj.com/site/about/guidelines.xhtml

		(*b*) Report category boundaries when continuous variables were categorized	7
		(*c*) If relevant, consider translating estimates of relative risk into absolute risk for a meaningful time period	n.a
Other analyses	17	Report other analyses done—eg analyses of subgroups and interactions, and sensitivity analyses	9
**Discussion**			
Key results	18	Summarise key results with reference to study objectives	11
Limitations	19	Discuss limitations of the study, taking into account sources of potential bias or imprecision. Discuss both direction and magnitude of any potential bias	12-13
Interpretation	20	Give a cautious overall interpretation of results considering objectives, limitations, multiplicity of analyses, results from similar studies, and other relevant evidence	11
Generalisability	21	Discuss the generalisability (external validity) of the study results	13
**Other information**			
Funding	22	Give the source of funding and the role of the funders for the present study and, if applicable, for the original study on which the present article is based	14

*Give information separately for exposed and unexposed groups.

Note: An Explanation and Elaboration article discusses each checklist item and gives methodological background and published examples of transparent reporting. The STROBE checklist is best used in conjunction with this article (freely available on the Web sites of PLoS Medicine at http://www.plosmedicine.org/, Annals of Internal Medicine at http://www.annals.org/, and Epidemiology at http://www.epidem.com/). Information on the STROBE Initiative is available at www.strobe-statement.org.

For peer review only

BMJ Open

Chronic diseases and multimorbidity among unemployed and employed persons in the Netherlands: a register-based cross-sectional study

Journal:	*BMJ Open*
Manuscript ID	bmjopen-2019-035037.R1
Article Type:	Original research
Date Submitted by the Author:	21-Apr-2020
Complete List of Authors:	Yildiz, Berivan; Erasmus Medical Center, Department of Public Health<br>Schuring, Merel; Erasmus MC, Department of Public Health<br>Knoef, Marike; Leiden University, Department of Economics<br>Burdorf, Alex; Erasmus MC, Department of Public Health
**Primary Subject Heading**:	Public health
Secondary Subject Heading:	Occupational and environmental medicine
Keywords:	PUBLIC HEALTH, EPIDEMIOLOGY, OCCUPATIONAL & INDUSTRIAL MEDICINE

SCHOLARONE™
Manuscripts

I, the Submitting Author has the right to grant and does grant on behalf of all authors of the Work (as defined in the below author licence), an exclusive licence and/or a non-exclusive licence for contributions from authors who are: i) UK Crown employees; ii) where BMJ has agreed a CC-BY licence shall apply, and/or iii) in accordance with the terms applicable for US Federal Government officers or employees acting as part of their official duties; on a worldwide, perpetual, irrevocable, royalty-free basis to BMJ Publishing Group Ltd ("BMJ") its licensees and where the relevant Journal is co-owned by BMJ to the co-owners of the Journal, to publish the Work in this journal and any other BMJ products and to exploit all rights, as set out in our licence.

The Submitting Author accepts and understands that any supply made under these terms is made by BMJ to the Submitting Author unless you are acting as an employee on behalf of your employer or a postgraduate student of an affiliated institution which is paying any applicable article publishing charge ("APC") for Open Access articles. Where the Submitting Author wishes to make the Work available on an Open Access basis (and intends to pay the relevant APC), the terms of reuse of such Open Access shall be governed by a Creative Commons licence – details of these licences and which Creative Commons licence will apply to this Work are set out in our licence referred to above.

Other than as permitted in any relevant BMJ Author's Self Archiving Policies, I confirm this Work has not been accepted for publication elsewhere, is not being considered for publication elsewhere and does not duplicate material already published. I confirm all authors consent to publication of this Work and authorise the granting of this licence.

For peer review only

Chronic diseases and multimorbidity among unemployed and employed persons in the Netherlands: a register-based cross-sectional study

Berivan Yildiz[1], MSc, Merel Schuring[1], PhD, Marike G. Knoef[2], PhD, Alex Burdorf[1], PhD

[1] Department of Public Health, Erasmus University Medical Center, Rotterdam, The Netherlands

[2] Department of Economics, Leiden University, Leiden, The Netherlands

Address of the corresponding author:

M. Schuring, Erasmus University Medical Center, Department of Public Health, P.O. Box 2040, 3000 CA Rotterdam, The Netherlands.

Email: m.schuring@erasmusmc.nl

Phone: 0031 10 703 89 93

Word count: 3758

For peer review only

ABSTRACT

Objectives: The first objective of this study was to describe the age specific prevalence of chronic diseases and multimorbidity among unemployed and employed persons. The second objective was to examine associations of employment status and sociodemographic characteristics with chronic diseases and multimorbidity.

Design: Data linkage of cross-sectional nation-wide registries on employment status, medication use and sociodemographic characteristics in 2016 was applied.

Setting: Register based data covering residents in the Netherlands.

Participants: 5,074,227 persons aged 18-65 years were selected with information on employment status, medication use and sociodemographic characteristics.

Outcome measures: Multiple logistic regression analysis and descriptive statistics were performed to examine associations of employment and sociodemographic characteristics with the prevalence of chronic diseases and multimorbidity. The age-specific prevalence of six common chronic diseases was described, and venn diagrams were applied for multimorbidity among unemployed and employed persons.

Results: Unemployed persons had a higher prevalence of psychological disorders (18.3% vs 5.4%), cardiovascular diseases (20.1% vs 8.9%), inflammatory diseases (24.5% vs 15.8%), and respiratory diseases (11.7% vs 6.5%) than employed persons. Unemployed persons were more likely to have one (OR 1.30 (1.29-1.31)), two (OR 1.74 (1.73-1.76)) and at least three chronic diseases (OR 2.59 (2.56-2.61)) than employed persons. Among unemployed persons, psychological disorders and inflammatory conditions increased with age but declined from middle age onwards, whereas a slight increase was observed among employed persons. Older persons, women, lower educated persons and migrants were more likely to have chronic diseases..

Conclusion: Large differences exist in the prevalence of chronic diseases and multimorbidity among unemployed and employed persons. The age specific prevalence follows a different pattern among employed and unemployed persons, with a relatively high prevalence of psychological disorders and inflammatory conditions among middle aged unemployed persons. Policy measures should focus more on promoting employment among unemployed persons with chronic diseases.

For peer review only - http://bmjopen.bmj.com/site/about/guidelines.xhtml

Strengths and limitations of this study

- This is the first study that describes the prevalence of chronic diseases and multimorbidity among unemployed and employed persons, using objective register-based data rather than self-reported health outcomes.
- A strength of this study is applying data linkage of nation-wide registries that capture the whole population, facilitating precise estimations of associations between health and employment, and offering us the possibility to investigate specific subgroups (age specific prevalence).
- Except for back pain and musculoskeletal disorders, this study investigated a broad range of chronic diseases such as cardiovascular diseases, psychological disorders, diabetes and respiratory diseases.
- Causal effects of having a chronic disease on employment status, or vice versa cannot be distinguished because of the use of cross-sectional data and the bidirectional nature of health and employment.

For peer review only

INTRODUCTION

The relationship between unemployment and health has been well established. (1, 2) In general, unemployed individuals have worse mental and physical health compared to employed individuals.(2-5) These health inequalities between employed and unemployed persons can be explained by two different hypotheses. First, according to the causation hypothesis, persons who become unemployed will deteriorate in health, whereas unemployed persons who enter paid employment will improve in health. (6) Second, the selection hypothesis describes that persons who leave paid employment already have lower levels of health before leaving employment compared to those who remain employed, whereas persons who enter paid employment already have a better health status before entering employment compared to persons who remain unemployed. (7)

Chronic diseases can affect an individual's employment status due to experienced functional limitations and a poor quality of life.(8) Two studies have shown that long term health conditions such as cardiovascular diseases and diabetes were associated with unemployment. (9, 10) In addition, a recent systematic review provided evidence that individuals with diabetes were more likely to be out of the labour force. (9) However, studies investigating chronic diseases and employment have mostly focused on a single disease, whereas many persons with a chronic disease are likely to suffer from multiple chronic diseases, especially among older age groups. There is increasing evidence that persons with multimorbidity - the co-occurrence of at least two chronic diseases within an individual - may be more likely to have poorer functional outcomes and thus may be also more often out of the labour market than those with a single chronic disease or no chronic disease. For instance, a study among Australian workers with multiple health problems showed that individuals with four or more health problems were far less likely to be employed compared to those with no health condition. (11) Another study among persons with back complaints found that the co-occurrence of cardiovascular diseases resulted in a 10-fold increased risk of unemployment compared to those with back complaints alone. (12)

So far, findings on the association of chronic diseases and multimorbidity with unemployment have been based mostly on self-reported health outcomes. Self-reported health outcomes are known to be vulnerable to reporting bias and justification bias. Therefore, a more objective approach is preferred in order to make more precise estimations of the prevalence of diseases. One way to objectively investigate the presence of chronic diseases is by using pharmacy data. (13) Pharmacy data provide a

For peer review only - http://bmjopen.bmj.com/site/about/guidelines.xhtml

reliable information source and often cover a large population. (14) Administrative databases such as drug prescription can be used to identify persons with chronic diseases. So far, only a few studies have used register-based data in order to investigate chronic diseases among unemployed persons. A Danish register-based study found a higher prevalence of mental disorders and cardiovascular disorders among unemployed persons receiving social benefits compared to employed persons. (15) In line with this, another register-based study showed that long-term unemployment was associated with a higher risk of antidepressant use. (16)

To our knowledge, none of the evidence on the association between multimorbidity and employment status has been based on register-based data. Therefore, the present study aimed to investigate the prevalence of chronic diseases and multimorbidity among unemployed and employed persons. The large register data enabled to investigate specific subgroups (e.g. age specific prevalences and associations). The second aim was to examine associations of employment status and sociodemographic characteristics with chronic diseases and multimorbidity. Nation-wide data from the Netherlands on drug prescription, employment status and sociodemographic characteristics in 2016 were used.

For peer review only

METHODS

Study population and design

Register data covering information on all Dutch residents in 2016 were used. Statistics Netherlands provided individual-level databases on demographics, education, labor market status and prescribed medication. All Dutch residents were pseudonymized using a personal unique number. Data registries were linked at the individual level using these pseudonymized numbers. No informed consent was needed for this study since authorized research institutes in the Netherlands are by law allowed to use pseudonymized register-based data for research purposes.

Individuals aged between 18 and 64 years with available information on employment status were selected (n=10,514,271). This selection captured individuals who were not eligible for exit from paid employment through statutory national retirement schemes. Due to the lack of nationwide education registers in the past, many older persons had missing data on educational level. Also, the current register only includes formal education obtained at institutes financed by government. Therefore, we excluded 31.9% of individuals with missing data on educational level (n=3,356,002). Within the population with available data on all sociodemographic characteristics (n=7,158,269), 4,566,644 persons were classified as employed and 507,583 persons were classified as unemployed. In total, 5,074,227 subjects were included in the present study.

Employment status

The database on social economic category per month (SECM) provided information on employment status of participants for each month in the year 2016. The main source of income for each consecutive month was used to classify persons as employed or unemployed. Individuals who were in paid employment or self-employed for at least 9 months in 2016 were classified as employed (n=4,566,644). Individuals who were out of the labour market and received either social benefits or unemployment benefits for at least 9 months were classified as unemployed (n=507,583).

Chronic diseases and multimorbidity

The database on medication use in 2016 (Medicijntab) provides information on purchased drugs that were reimbursed by the health care insurances. The drugs were identified using the World Health Organization Anatomical Therapeutic Chemical (ATC) classification codes (17). In line with the study of

For peer review only - http://bmjopen.bmj.com/site/about/guidelines.xhtml

Huber et al, specific chronic diseases were identified based on these ATC-codes. (13) For instance, psychological disorders were identified by ATC-codes that were assigned to drugs such as antidepressants and anxiolytics, whereas inflammatory conditions were identified by the ATC-code that was assigned to non-steroidal anti-inflammatory drugs (NSAID's) (Supplementary Table 1).

The presence of a specific chronic disease was dichotomized into having or not having a chronic disease. Multimorbidity was investigated as 1) the number of chronic diseases and 2) the combinations of four common chronic diseases with the highest prevalence in the study population. For the first approach of multimorbidity, the total number of chronic diseases was computed for each participant, based on 21 different chronic diseases that could be identified by medication prescription. (13) This measure of multimorbidity was categorized into four groups: no chronic disease, one chronic disease, two chronic diseases, and at least three chronic diseases.. For the second approach, we used the following four chronic diseases to describe their co-occurrence: cardiovascular diseases, psychological disorders, inflammatory conditions, and respiratory diseases.

Sociodemographic variables

The databases on sociodemographic characteristics provide information on age, gender, education and migration background. A dichotomous variable was computed for employment status (employed vs. unemployed). Educational level was categorized into three educational groups: high (higher vocational training or university), intermediate (higher secondary and intermediate vocational training) and low education (pre-primary education, primary education, and lower secondary education). Age was categorized into four age groups (18-30, 30-45, 45-55, 55-65). Migration background was categorized as native Dutch, Moroccan, Turkish, Surinamese and Antillean, other Western, and other non-Western.

Analyses

Descriptive statistics were used to describe the prevalence of chronic diseases and multimorbidity among employed and unemployed persons. The association of sociodemographic characteristics (age, sex, education, and ethnic background) and employment status with (multiple) chronic diseases was examined using multiple logistic regression analysis in the total study population (employed and unemployed persons). Separate logistic regression analyses were done for each number of chronic diseases (dependent variable): i) one chronic disease ii) two chronic diseases and iii) three or more chronic diseases. In these analyses, having no chronic diseases was used as the reference category.

For peer review only - http://bmjopen.bmj.com/site/about/guidelines.xhtml

Logistic regression analyses were adjusted for age, sex, educational level and migration background. The association between employment status and multimorbidity stratified by age was also investigated. To test for possible selection bias, sensitivity analyses were performed by including individuals with missing data on educational level.

The prevalence of multimorbidity was also described as the proportion of individuals with all potential combinations of four exclusive chronic diseases. All combinations of co-occurrence between the four chronic diseases were presented in a venn diagram for employed and unemployed persons. The age-specific prevalence of these four chronic diseases among unemployed and employed persons was presented. In order to distinguish specific conditions within a chronic disease, the age-specific prevalence of specific medicines was also investigated for cardiovascular diseases and psychological disorders. The latter was not investigated for the other chronic diseases because less specific medicines could be distinguished within the other chronic disease.

Patient and public involvement

No patients were involved.

For peer review only

For peer review only - http://bmjopen.bmj.com/site/about/guidelines.xhtml

RESULTS

Unemployed persons were more often older than 45 years (58.2%), female (54.5%), lower educated (51.8%) and from non-Dutch origin (48.1%) compared to employed persons (19.4%). Differences in the prevalence of chronic diseases between unemployed and employed persons were highest for psychological disorders. Compared to employed persons, unemployed persons had a higher prevalence of psychological disorders (18.3% vs 5.4%), cardiovascular diseases (20.1% vs 8.9%), inflammatory conditions (24.5% vs 15.8%), psychotic illness (6.2% vs 0.8%), respiratory diseases (11.7% vs 6.5%) and diabetes (7.2% vs 2.0%). (Table 1)

Table 1. Characteristics of the study population by employment status.

	**Unemployed (n=507,583)**	**Employed (n=4,566,644)**
Age	n (%)	n (%)
18-30	54,807 (10.8)	1,223,211 (26.8)
30-45	157,238 (31.0)	1,808,274 (39.6)
45-55	147,865 (29.1)	1,033,090 (22.6)
55-65	147,673 (29.1)	502,069 (11.0)
Sex		
Male	230,856 (45.4)	2,405,740 (52.7)
Female	276,727 (54.5)	2,160,904 (47.3)
Educational level		
High	73,893 (14.6)	2,022,717 (44.3)
Middle	170,857 (33.7)	1,906,830 (41.8)
Low	262,833 (51.8)	637,097 (14.0)
Migration background		
Native Dutch	263,196 (51.9)	3,680,071 (80.6)
Moroccan	35,441 (7.0)	76,841 (1.7)
Turkish	27,131 (5.3)	97,716 (2.1)
Surinamese & Antillean	40,446 (8.0)	145,478 (2.3)
Other Western	37,420 (7.4)	311,005 (6.8)
Other non-Western	103,949 (20.5)	255,533 (5.6)
Chronic diseases		
Inflammatory conditions	124,411 (24.5)	721,304 (15.8)
Cardiovascular diseases	101,917 (20.1)	405,200 (8.9)
Psychological disorders	92,956 (18.3)	248,520 (5.4)
Respiratory diseases	59,557 (11.7)	296,817 (6.5)
Diabetes	36,662 (7.2)	89,382 (2.0)
Psychotic illness	31,308 (6.2)	34,377 (0.8)
Number of chronic diseases		
0	193,412 (38.1)	2,877,313 (63.0)
1	118,688 (23.4)	1,007,275 (22.1)
2	79,719 (15.7)	394,396 (8.6)

For peer review only - http://bmjopen.bmj.com/site/about/guidelines.xhtml

≥3	115,764 (22.8)	287,660 (6.3)

The prevalence of multimorbidity was also higher for unemployed persons compared to employed persons. The co-occurrence of both psychological disorders and inflammatory conditions was higher among unemployed persons (3.6%+0.9%+1.2%+0.5%=6.2%) than among employed persons (1.0%+0.2%+0.2%+0.1%=1.5%). In addition, the co-occurrence of cardiovascular diseases and inflammatory conditions was higher among unemployed persons (5.5%) compared to employed persons (2.1%). The prevalence of having both cardiovascular diseases and psychological disorders was 4.9% among unemployed persons compared to 0.9% among employed persons. (Figure 1, Supplementary Table 2)

At all ages, unemployed individuals had a higher prevalence of all four chronic diseases compared to employed individuals. The prevalence of psychological disorders increased with age followed by a decrease from middle age onwards among unemployed persons, whereas a slight increase was observed among employed persons. The same pattern was observed for inflammatory conditions. The prevalence of cardiovascular diseases and respiratory diseases increased with age among both unemployed and employed persons. (Figure 2)

Among unemployed persons, the use of antidepressants, anxiolytics and, hypnotics and sedatives was highest at middle age. The prevalence of antidepressants was higher than the use of anxiolytics, hypnotics and sedatives among unemployed persons for all age groups. The use of antithrombotic and cardiac agents, beta blockers and angiotensin-convertin-enzyme (ACE) inhibitors, and diuretics and calcium-channel blockers was highestamong older unemployed and employed persons. The prevalence of beta blockers and ACE inhibitors, and antithrombotic agents was higher than diuretics and calcium-channel blockers, and cardiac agents among unemployed persons of all age groups. (Supplementary Figure 1)

Unemployed persons were more likely to have (multiple) chronic diseases compared to employed persons. Unemployed persons were more likely to have one (OR 1.30, 95% CI 1.29-1.31), two (OR 1.74, 95% CI 1.73-1.76) and at least three chronic diseases (OR 2.59, 95% CI 2.56-2.61) than employed persons. Among unemployed persons, 23% had at least three chronic diseases compared to 6% among

For peer review only - http://bmjopen.bmj.com/site/about/guidelines.xhtml

employed persons. Women (OR 1.49, 95% CI 1.49-1.50), older individuals (OR 1.32 - 2.27), middle and low educated persons (OR 1.32 – 1.49), and non-western migrants (OR 1.01 - 1.34) were also more likely to have a chronic disease. In addition, women (OR 1.61, 95% CI 1.60-1.63), older persons (OR 2.72 - 17.08), lower educated persons (OR 2.11 - 3.32) and non-western migrants (OR 1.02 - 2.03) were also more likely to have multiple (≥3) chronic diseases (Table 2). Comparable results were found in sensitivity analyses on the total study population with inclusion of individuals who had missing data (n=7,576,196) on educational level.

Within all age groups, unemployed persons had a higher risk of having one, two and at least three chronic diseases than employed persons. Especially, among persons aged 18-30 years, unemployed persons were more likely to have one (OR 1.65, 95% CI 1.61-1.69), two (OR 2.84 (95% CI 2.75-2.93) and at least three (OR 5.20, 95% CI 4.99-5.42) chronic diseases compared to employed persons. These effect estimates were lower among older age groups. (Supplementary Table 3)

For peer review only

For peer review only - http://bmjopen.bmj.com/site/about/guidelines.xhtml

1 **Table 2.** The association of sociodemographic characteristics with the number of chronic diseases in the
2 total population (n=5,074,227).

	One chronic disease*		Two chronic diseases *		At least three chronic diseases*	
	**n (%)**	**OR (95% CI)**	**n (%)**	**OR (95% CI)**	**n (%)**	**OR (95% CI)**
Employment status						
Employed (n=4,566,644)	1,007,275 (22.1%)	1	394,396 (8.6%)	1	287,660 (6.3%)	1
Unemployed (n=507,583)	118,688 (23.4%)	1.30 (1.29-1.31)	79,719 (15.7%)	1.74 (1.73-1.76)	115,764 (22.8%)	2.59 (2.56-2.61)
Gender						
Male (n=2,636,596)	524,931 (19.9%)	1	214,039 (8.1%)	1	179,540 (6.8%)	1
Female (n=2,437,631)	601,032 (24.7%)	1.49 (1.49 - 1.50)	260,076 (10.7%)	1.60 (1.59-1.61)	223,884 (9.2%)	1.61 (1.60-1.63)
Age						
18-30 (n=1,278,018)	249,361 (19.5%)	1	66,065 (5.2%)	1	25,443 (2.0%)	1
30-45 (n=1,965,512)	435,651 (22.2%)	1.32 (1.31-1.33)	154,922 (7.9%)	1.78 (1.77-1.80)	92,888 (4.7%)	2.72 (2.68-2.76)
45-55 (n=1,180,955)	286,880 (24.3%)	1.80 (1.79-1.81)	146,145 (12.4%)	3.38 (3.35-3.41)	143,542 (12.2%)	8.00 (7.88-8.11)
55-65 (n=649,742)	154,071 (23.7%)	2.27 (2.26-2.29)	106,983 (16.5%)	5.62 (5.55-5.68)	141,551 (21.8%)	17.08 (16.83-17.33)
Educational level						
High (n=2,096,610)	436,035 (20.8%)	1	150,868 (7.2%)	1	89,730 (4.3%)	1
Middle (n=2,077,687)	478,089 (23.0%)	1.32 (1.31-1.32)	202,938 (9.8%)	1.62 (1.60-1.63)	162,416 (7.8%)	2.11 (2.09-2.13)
Low (n=899,930)	211,839 (23.5%)	1.49 (1.48-1.50)	120,309 (13.4%)	2.06 (2.04-2.07)	151,278 (16.8%)	3.32 (3.29-3.36)
Migration background						
Dutch (n=3,943,267)	869,203 (22.0%)	1	356,489 (9.0%)	1	286,169 (7.3%)	1
Moroccan (n=112,282)	27,621 (24.6%)	1.26 (1.25-1.28)	13,278 (11.8%)	1.38 (1.35-1.41)	13,840 (12.3%)	1.49 (1.45-1.52)
Turkish (n=124,847)	30,208 (24.2%)	1.34 (1.32-1.36)	14,818 (11.9%)	1.58 (1.55-1.61)	17,208 (13.8%)	2.03 (1.99-2.07)
Surinamese & Antillean (n=185,924)	42,837 (23.0%)	1.10 (1.09-1.11)	20,541 (11.0%)	1.19 (1.17-1.21)	22,149 (11.9%)	1.39 (1.37-1.41)
Other Western (n=348,425)	73,959 (21.2%)	0.91 (0.90-0.91)	30,235 (8.7%)	0.88 (0.87-0.89)	25,345 (7.3%)	0.87 (0.85-0.88)
Other Non-Western (n=359,482)	82,135 (22.8%)	1.01 (1.00-1.02)	38,754 (10.8%)	1.01 (0.99-1.02)	38,713 (10.8%)	1.02 (1.01-1.03)

3 *persons having no chronic diseases constituted the reference group.

For peer review only

DISCUSSION

In this large register-based study, unemployed persons had a higher prevalence of cardiovascular diseases, psychological disorders, inflammatory conditions, respiratory diseases and multimorbidity compared to employed persons. Between unemployed and employed persons, the largest differences were observed for cardiovascular diseases and psychological disorders. The prevalence of psychological disorders and inflammatory conditions was highest among unemployed persons in the middle age group. Women, older individuals, lower educated persons and non-western migrants were more likely to have one chronic disease as well as multiple chronic diseases. Among younger persons (18-30 years), a stronger association between chronic disease and unemployment was found compared to higher age groups.

The higher prevalence of chronic diseases among unemployed persons in the current study is in line with other studies that unemployed persons have a poorer mental and physical health status. (2, 5, 18) For instance, unemployed persons had high risks of common mental disorders such as depression. (19-21) Our study added to the current literature by comparing the age-specific prevalence of chronic diseases between unemployed and employed persons. Between unemployed and employed persons, the largest differences were observed for cardiovascular diseases and psychological disorders. Although the overall prevalence of cardiovascular diseases was much higher among unemployed persons, the age-specific patterns showed small differences, indicating that the higher age among unemployed persons was largely responsible for the higher prevalence of cardiovascular diseases.

A remarkable finding was the different pattern of the age-specific prevalence of psychological disorders between unemployed and employed persons. Among both employed and unemployed persons, the prevalence of psychological disorders increased with age. However, this increase was more profound among unemployed persons with a peak around middle age. This pattern among unemployed persons can be explained by studies arguing that before and after middle age, individuals tend to suffer less from unemployment compared to persons of middle age. (22) Persons of middle age often have family responsibilities, increasing the financial importance of a job, whereas younger and older persons experience less financial pressure and thus less psychological distress due to unemployment. (23) The other way around, it can also be that the combination of family responsibilities and work lead to pressure and cause both health problems as well as unemployment. Furthermore, it has also been

For peer review only - http://bmjopen.bmj.com/site/about/guidelines.xhtml

hypothesized that persons of middle age are more likely to aim for a successful career which leads to employment being more important for their mental health than it is for older persons who are almost finishing their careers, and for younger persons who have recently entered paid employment. (24) The age-specific prevalence of antidepressants use, anxiolytics, and hypnotics and sedatives among unemployed persons confirms this theory by showing the highest prevalence at middle age and a lower prevalence at younger and older ages.

It was checked whether the decline in the prevalence of use of antidepressants, anxiolytics, hypnotics and sedatives after the age of 50 would be different among persons receiving a disability benefit. Namely, it might be possible that older unemployed persons with chronic diseases are more likely to receive disability benefits rather than unemployment or social benefits, and therefore the age-specific prevalence of these medicines declines among unemployed persons. However, also among persons with disability benefits, la decline was observed from middle age onwards for these medicines (results not shown).

Another interesting finding was an increase in the prevalence of inflammatory conditions with increasing age followed by a decrease from middle age onwards among unemployed persons. This finding can be explained by the medicines NSAID's that have been used to identify inflammatory conditions as a chronic disease in the present study. NSAID's are pain killers with anti-inflammatory effects and are known to cause serious adverse effects. (25) Therefore, NSAID's are cautiously or not prescribed among individuals aged 60 years or older, who suffer from cardiovascular diseases and already use other medicines for other (chronic) health conditions. (26) In the present study, the prevalence of cardiovascular diseases and multimorbidity was higher among unemployed persons compared to employed persons. Thus, it may be that NSAID's are less prescribed among older persons because of multimorbidity with cardiovascular disorders and associated polypharmacy – the use of multiple medicines. Therefore, the higher prevalence of multimorbidity among unemployed persons may explain the decrease in the prevalence of inflammatory conditions with increasing age.

Unemployed persons had a higher prevalence of multimorbidity than employed persons. It is likely that the healthy worker selection process is more prominent among persons with multiple diseases than single diseases. (27) According to the causation mechanism, it could also be that persons who become unemployed will deteriorate in health. Underlying mechanisms have been proposed by Jahoda who

For peer review only - http://bmjopen.bmj.com/site/about/guidelines.xhtml

posits that unemployed persons may lack five latent functions usually observed among employed persons such as a time structure, being useful, social contacts, social status and being active. (28) The latter causation mechanism suggests that it is important that next to addressing chronic diseases, these psychosocial factors are targeted as well by interventions, in order to improve the health of unemployed persons. Improving health and employment opportunities for persons with chronic diseases is also important in the light of an aging workforce with an expected increase of multimorbidity during the next decades.

The strength of the present study is the use of register-based data, which is a more objective method to investigate the association between health and unemployment. In earlier studies, the relationship between health and unemployment has often been examined using self-reported outcomes of health and disability. (18) However, a major concern of self-reported health outcomes is that unemployed individuals may over-report their level of disability or work limitations to justify that they are not in paid employment. (29) In the current study, this problem has been minimized using register-based data on medication use that has been collected independently of the study. Another strength of the current study is that our register-based data capture the whole Dutch population and therefore the data provide statistical power to investigate age-specific prevalences. This facilitates precise estimations of associations between health and employment. Lastly, the use of register-based data is less expensive since no additional efforts have to be made for data collection and no concerns are present about health-related non-response.

Register-based data also have some limitations as the register only includes individuals who fulfill three criteria: 1) they are considered to need a particular drug by their general practitioner or specialist, 2) they purchase the prescribed medicine at the pharmacy, and 3) the costs of the medicines are reimbursed by health insurances. For instance, persons with psychological disorders who are treated with a cognitive behavioral therapy rather than medication are not included in our analysis, and this may lead to an underestimation of persons with psychological disorders. Moreover, although a broad range of chronic diseases has been investigated in this study, several conditions that are associated with unemployment have not been included, such as back pain or musculoskeletal disorders. In the current study, it was not possible to identify these chronic conditions by the use of medication data. For instance, medication that is prescribed for back pain includes over the counter pain killers such as paracetamol or ibuprofen. However, since pain killers are used for various forms of bodily pain and no

For peer review only - http://bmjopen.bmj.com/site/about/guidelines.xhtml

information was available regarding the reason of prescription, it was not possible to identify these health conditions. Nevertheless, it is possible that inflammatory conditions include musculoskeletal problems, as NSAID´s are a common treatment. (26) Conditions such as back pain and musculoskeletal disorders are known to lead to exit from paid employment and therefore should be investigated among unemployed persons in future studies.(12)

A second limitation of this study was that the cross-sectional design did not allow to gain insight into the bi-directional association between unemployment and health. However, this study provided pivotal evidence for the large differences in the prevalence of chronic diseases between unemployed and employed persons. Longitudinal or (quasi-) experimental studies are needed to further elaborate how chronic diseases lead to unemployment, and unemployment may result in chronic diseases and multimorbidity. A third limitation of this study relates to the selection of the study population of unemployed persons. Since the criteria for unemployment was defined as being unemployed for at least 9 months during a period of one year, our results and conclusions mainly apply to persons who are long-term unemployed. It may be that associations found in this study are less strong among short-term unemployed persons as they may have less health problems. Lastly, a limitation of the current study was the exclusion of individuals with missing data on educational level. Unemployed persons in this study more often had a lower educational level than employed person. Since there is an association between lower educational level and poorer health status, it was important to adjust for educational level in several statistical analyses. The sensitivity analysis showed comparable results in the total population and the population with educational information, indicating that education was most likely missing at random.

This study showed that health inequalities exist between unemployed and employed persons. Specifically, among the younger age group, a strong association of chronic diseases and multimorbidity with unemployment was found. Several studies have shown the beneficial effects of employment on health. (30, 31) According to these studies, interventions that can support unemployed persons with chronic diseases are needed to improve employment opportunities and thus health. In order to reduce health inequalities between unemployed and employed persons, it is therefore important that re-integration policies will focus more on promoting employment among unemployed persons with chronic diseases.

For peer review only - http://bmjopen.bmj.com/site/about/guidelines.xhtml

In conclusion, the current study showed that unemployed persons more often have chronic diseases and multimorbidity than employed persons. The age specific prevalence follows a different pattern among employed and unemployed persons, with a relatively high prevalence of psychological disorders and inflammatory conditions among middle aged unemployed persons. Policy measures are needed to improve health and promote employment among unemployed persons.

Acknowledgements

-

Contributors

BY and MS prepared the data. BY performed the statistical analysis, drafted and revised the article. MS, MGL and AB participated in the analyses. MS, MGK and AB critically reviewed the manuscript. All authors approved the final version.

Funding

This study was funded by ZonMw (The Netherlands Organisation for Health Research and Development).

Competing interests

The authors declare no conflict of interest.

Patient consent for publication

Not required.

Data sharing statement

No data is available.

For peer review only

References

1. Janlert U. Unemployment as a disease and diseases of the unemployed. Scand J Work Environ Health. 1997;23 Suppl 3:79-83.
2. McKee-Ryan F, Song Z, Wanberg CR, Kinicki AJ. Psychological and physical well-being during unemployment: a meta-analytic study. J Appl Psychol. 2005;90(1):53-76.
3. Bartley M, Sacker A, Clarke P. Employment status, employment conditions, and limiting illness: prospective evidence from the British household panel survey 1991-2001. J Epidemiol Community Health. 2004;58(6):501-6.
4. Wanberg CR. The individual experience of unemployment. Annu Rev Psychol. 2012;63:369-96.
5. Roos E, Lahelma E, Saastamoinen P, Elstad JI. The association of employment status and family status with health among women and men in four Nordic countries. Scand J Public Health. 2005;33(4):250-60.
6. Schuring M, Robroek SJ, Lingsma HF, Burdorf A. Educational differences in trajectories of self-rated health before, during, and after entering or leaving paid employment in the European workforce. Scand J Work Environ Health. 2015;41(5):441-50.
7. Thomas C, Benzeval M, Stansfeld SA. Employment transitions and mental health: an analysis from the British household panel survey. J Epidemiol Community Health. 2005;59(3):243-9.
8. Varekamp I, van Dijk FJH, Kroll LE. Workers with a chronic disease and work disability. Bundesgesundheitsblatt - Gesundheitsforschung - Gesundheitsschutz. 2013;56(3):406-14.
9. Pedron S, Emmert-Fees K, Laxy M, Schwettmann L. The impact of diabetes on labour market participation: a systematic review of results and methods. BMC public health. 2019;19(1):25-.
10. Leal J, Luengo-Fernandez R, Gray A, Petersen S, Rayner M. Economic burden of cardiovascular diseases in the enlarged European Union. Eur Heart J. 2006;27(13):1610-9.
11. Schofield DJ, Callander EJ, Shrestha RN, Passey ME, Percival R, Kelly SJ. Multiple chronic health conditions and their link with labour force participation and economic status. PLoS One. 2013;8(11):e79108.
12. Schofield DJ, Callander EJ, Shrestha RN, Passey ME, Percival R, Kelly SJ. Association between co-morbidities and labour force participation amongst persons with back problems. Pain. 2012;153(10):2068-72.
13. Huber CA, Szucs TD, Rapold R, Reich O. Identifying patients with chronic conditions using pharmacy data in Switzerland: an updated mapping approach to the classification of medications. BMC Public Health. 2013;13:1030.
14. Zhan C, Miller M. Administrative data based patient safety research: a critical review. Quality & safety in health care. 2003;12(Suppl 2):ii58-ii63.
15. Andersen I, Brønnum-Hansen H, Kriegbaum M, Hougaard CØ, Hansen FK, Diderichsen F. Increasing illness among people out of labor market–A Danish register-based study. Social Science & Medicine. 2016;156:21-8.
16. Virtanen M, Kivimaki M, Ferrie JE, Elovainio M, Honkonen T, Pentti J, et al. Temporary employment and antidepressant medication: a register linkage study. J Psychiatr Res. 2008;42(3):221-9.
17. World Health O. WHO Collaborating Centre for Drug Statistics Methodology: ATC classification index with DDDs and Guidelines for ATC classification and DDD assignment. Oslo, Norway: Norwegian Institute of Public Health. 2006.

For peer review only - http://bmjopen.bmj.com/site/about/guidelines.xhtml

18. Luo J, Qu Z, Rockett I, Zhang X. Employment status and self-rated health in north-western China. Public Health. 2010;124(3):174-9.
19. Stankunas M, Kalediene R, Starkuviene S, Kapustinskiene V. Duration of unemployment and depression: a cross-sectional survey in Lithuania. BMC Public Health. 2006;6:174.
20. Khlat M, Sermet C, Le Pape A. Increased prevalence of depression, smoking, heavy drinking and use of psycho-active drugs among unemployed men in France. European journal of epidemiology. 2004;19(5):445-51.
21. Ford E, Clark C, McManus S, Harris J, Jenkins R, Bebbington P, et al. Common mental disorders, unemployment and welfare benefits in England. Public health. 2010;124(12):675-81.
22. Broomhall HS, Winefield AH. A comparison of the affective well-being of young and middle-aged unemployed men matched for length of unemployment. British Journal of Medical Psychology. 1990;63(1):43-52.
23. Jackson PR, Warr PB. Unemployment and psychological ill-health: the moderating role of duration and age. Psychological Medicine. 1984;14(3):605-14.
24. Lahelma E. Unemployment, re-employment and mental well-being. A panel survey of industrial jobseekers in Finland. Scandinavian Journal of Social Medicine, Supplement. 1989;17(43):1-170.
25. Sostres C, Gargallo CJ, Arroyo MT, Lanas A. Adverse effects of non-steroidal anti-inflammatory drugs (NSAIDs, aspirin and coxibs) on upper gastrointestinal tract. Best Practice & Research Clinical Gastroenterology. 2010;24(2):121-32.
26. Atchison JW, Herndon CM, Rusie E. NSAIDs for musculoskeletal pain management:current perspectives and novel strategies to improve safety. J Manag Care Pharm. 2013;19(9 Suppl A):S3-19.
27. Li CY, Sung FC. A review of the healthy worker effect in occupational epidemiology. Occup Med (Lond). 1999;49(4):225-9.
28. Jahoda M. Employment and Unemployment: Cambridge University Press; 1982.
29. Bound J. Self-Reported Versus Objective Measures of Health in Retirement Models. The Journal of Human Resources. 1991;26(1):106-38.
30. Schuring M, Robroek SJ, Burdorf A. The benefits of paid employment among persons with common mental health problems: evidence for the selection and causation mechanism. Scand J Work Environ Health. 2017;43(6):540-9.
31. van der Noordt M, H IJ, Droomers M, Proper KI. Health effects of employment: a systematic review of prospective studies. Occup Environ Med. 2014;71(10):730-6.

For peer review only - http://bmjopen.bmj.com/site/about/guidelines.xhtml

Figure 1. Multimorbidity of cardiovascular diseases (CVD), psychological disorders (PD), inflammatory conditions (IC) and respiratory diseases (RD) among unemployed (n=507,583) and employed persons (n=4,566,644).

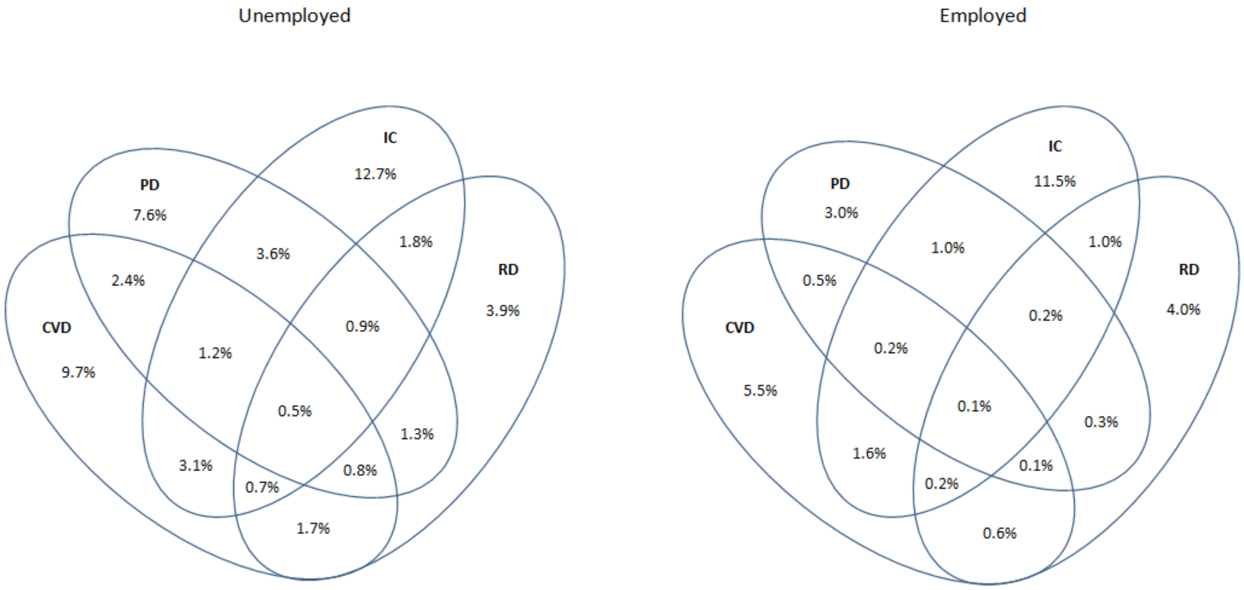

Figure 2. Prevalence of four chronic diseases by age among unemployed (n=507,583) and employed (n=4,566,644) persons in 2016.

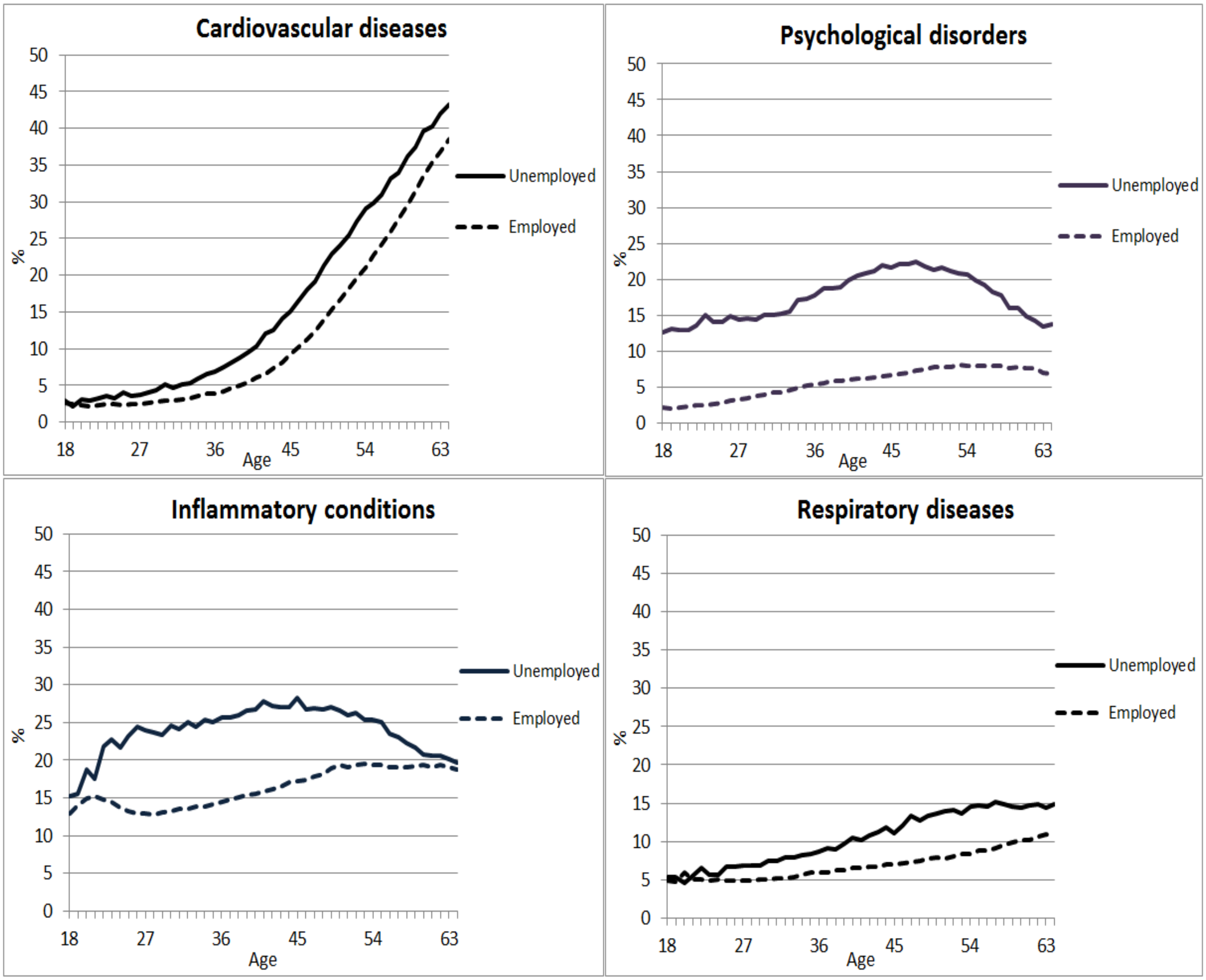

Supplementary Table 1. Description of ATC-codes used to identify chronic diseases among unemployed (n=507,583) and employed (n=4,566,644) persons.

Chronic disease	ATC-code	Medication class	Prevalence (%) Unemployed	Prevalence (%) Employed
**Cardiovascular disease**	B01A	Antithrombotic agents	8.5	3.2
	C01	Cardiac agents	1.9	0.7
	C03A	Low-ceiling drugs	3.8	1.5
	C08	Calcium channel blockers	5.4	1.9
	C07	Beta blocking agents	9.6	3.7
	C09A, C09B	ACE inhibitors	7.0	2.8
**Psychological disorders**	N05B	Anxiolytics	6.0	0.9
	N05C	Hypnotics and sedatives	3.4	0.4
	N06A	Antidepressants	14.4	5.0
**Inflammatory and antirheumatic products**	M01A	Anti-inflammatory and antirheumatic products, non-steroids	24.5	15.8
**Respiratory diseases**	R03A, R03C	Adrenergics (inhalants)	10.5	5.8
	R03B, R03D	Other drugs for obstructive airway diseases	4.8	2.3
**Psychotic illness**	N05A	Antipsychotics	6.2	0.8
**Diabetes mellitus**	A10A	Insulins and analogues	2.5	0.8
	A10B	Blood glucose lowering drugs	6.4	1.5

For peer review only

Supplementary Table 2. Combinations and prevalence of multimorbidity of four chronic diseases among unemployed (n=507,583) and employed (n=4,566,644) persons.

**Combination**	**Chronic disease**				**Prevalence**	
	**Cardiovascular diseases (CVD)**	**Psychological disorders (PD)**	**Inflammatory conditions (IC)**	**Respiratory diseases (RD)**	**N (%) Unemployed**	**N (%) Employed**
**1**	1	0	0	0	49,124 (9.7)	248,948 (5.5)
**2**	0	1	0	0	38,549 (7.6)	137,314 (3.0)
**3**	0	0	1	0	64,535 (12.7)	524,452 (11.5)
**4**	0	0	0	1	19,832 (3.9)	181,148 (4.0)
**5**	1	0	0	1	8,611 (1.7)	28,834 (0.6)
**6**	1	0	1	0	15,533 (3.1)	72,591 (1.6)
**7**	1	1	0	0	12,046 (2.4)	25,049 (0.5)
**8**	0	1	1	0	18,098 (3.6)	45,499 (1.0)
**9**	0	0	1	1	9,124 (1.8)	46,741 (1.0)
**10**	0	1	0	1	6,699 (1.3)	14,651 (0.3)
**11**	1	1	1	0	5,995 (1.2)	11,376 (0.2)
**12**	0	1	1	1	4,683 (0.9)	7,041 (0.2)
**13**	1	1	0	1	4,165 (0.8)	4,798 (0.1)
**14**	1	0	1	1	3,722 (0.7)	10,812 (0.2)
**15**	1	1	1	1	2,721 (0.5)	2,792 (0.1)

For peer review only

Supplementary Table 3. The association of employment status with multimorbidity stratified by age.

	Chronic diseases		
	**1**	**2**	**≥3**
	**OR (95% CI)**		
Employment status (n=5,074,227)			
Employed	1	1	1
Unemployed	1.30 (1.29-1.31)	1.74 (1.73-1.76)	2.59 (2.56-2.61)
Age 18-30 (n=1,278,018)			
Employed	1	1	1
Unemployed	1.65 (1.61-1.69)	2.84 (2.75-2.93)	5.20 (4.99-5.42)
Age 30-45 (n=1,965,512)			
Employed	1	1	1
Unemployed	1.41 (1.39-1.43)	2.12 (2.09-2.16)	3.52 (3.45-3.59)
Age 45-55 (n=1,180,955)			
Employed	1	1	1
Unemployed	1.22 (1.20-1.23)	1.64 (1.61-1.67)	2.66 (2.62-2.70)
Age 55-65 (n=649,742)			
Employed	1	1	1
Unemployed	1.09 (1.07-1.11)	1.29 (1.26-1.31)	1.78 (1.76-1.81)

Logistic regression analyses were adjusted for sex, educational level and migration background.

For peer review only

Supplementary Figure 1. Prevalence of medicines for psychological disorders (a and b) and cardiovascular diseases (c and d) by age among unemployed (n=507,583) and employed (n=4,566,644) persons in 2016.

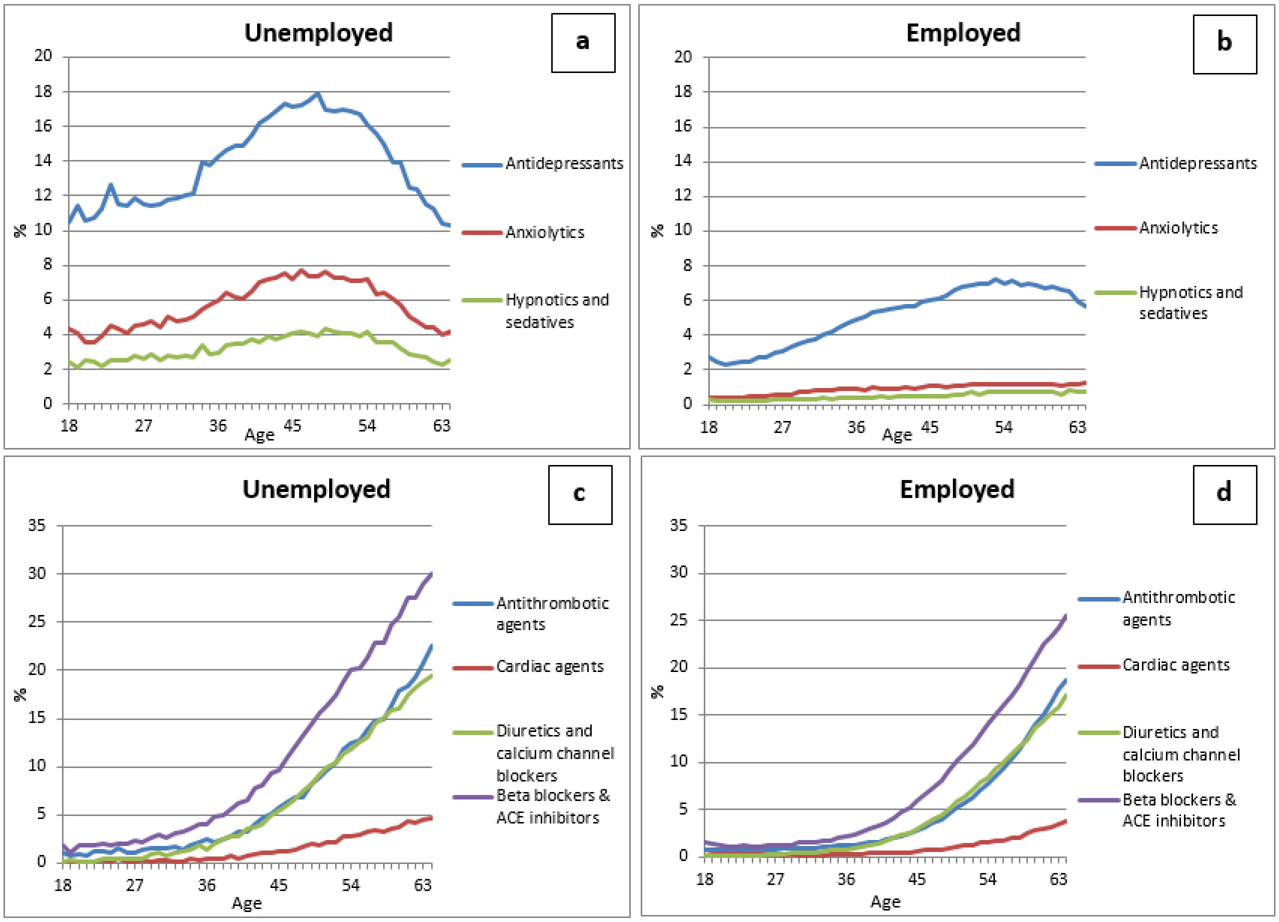

only

STROBE Statement—Checklist of items that should be included in reports of ***cross-sectional studies***

	Item No	Recommendation	Page No
**Title and abstract**	1	(*a*) Indicate the study's design with a commonly used term in the title or the abstract	0/1
		(*b*) Provide in the abstract an informative and balanced summary of what was done and what was found	1
**Introduction**			
Background/rationale	2	Explain the scientific background and rationale for the investigation being reported	3
Objectives	3	State specific objectives, including any prespecified hypotheses	4
**Methods**			
Study design	4	Present key elements of study design early in the paper	5
Setting	5	Describe the setting, locations, and relevant dates, including periods of recruitment, exposure, follow-up, and data collection	5
Participants	6	(*a*) Give the eligibility criteria, and the sources and methods of selection of participants	5
Variables	7	Clearly define all outcomes, exposures, predictors, potential confounders, and effect modifiers. Give diagnostic criteria, if applicable	5/6
Data sources/ measurement	8*	For each variable of interest, give sources of data and details of methods of assessment (measurement). Describe comparability of assessment methods if there is more than one group	*6*
Bias	9	Describe any efforts to address potential sources of bias	6
Study size	10	Explain how the study size was arrived at	5
Quantitative variables	11	Explain how quantitative variables were handled in the analyses. If applicable, describe which groupings were chosen and why	6
Statistical methods	12	(*a*) Describe all statistical methods, including those used to control for confounding	6
		(*b*) Describe any methods used to examine subgroups and interactions	6
		(*c*) Explain how missing data were addressed	6
		(*d*) If applicable, describe analytical methods taking account of sampling strategy	n.a
		(*e*) Describe any sensitivity analyses	6
**Results**			
Participants	13*	(a) Report numbers of individuals at each stage of study—eg numbers potentially eligible, examined for eligibility, confirmed eligible, included in the study, completing follow-up, and analysed	n.a/ 7
		(b) Give reasons for non-participation at each stage	6
		(c) Consider use of a flow diagram	
Descriptive data	14*	(a) Give characteristics of study participants (eg demographic, clinical, social) and information on exposures and potential confounders	7
		(b) Indicate number of participants with missing data for each variable of interest	5
Outcome data	15*	Report numbers of outcome events or summary measures	7-9
Main results	16	(*a*) Give unadjusted estimates and, if applicable, confounder-adjusted estimates and their precision (eg, 95% confidence interval). Make clear which confounders were adjusted for and why they were included	8-9

For peer review only

		(*b*) Report category boundaries when continuous variables were categorized	7
		(*c*) If relevant, consider translating estimates of relative risk into absolute risk for a meaningful time period	n.a
Other analyses	17	Report other analyses done—eg analyses of subgroups and interactions, and sensitivity analyses	9
**Discussion**			
Key results	18	Summarise key results with reference to study objectives	11
Limitations	19	Discuss limitations of the study, taking into account sources of potential bias or imprecision. Discuss both direction and magnitude of any potential bias	12-13
Interpretation	20	Give a cautious overall interpretation of results considering objectives, limitations, multiplicity of analyses, results from similar studies, and other relevant evidence	11
Generalisability	21	Discuss the generalisability (external validity) of the study results	13
**Other information**			
Funding	22	Give the source of funding and the role of the funders for the present study and, if applicable, for the original study on which the present article is based	14

*Give information separately for exposed and unexposed groups.

Note: An Explanation and Elaboration article discusses each checklist item and gives methodological background and published examples of transparent reporting. The STROBE checklist is best used in conjunction with this article (freely available on the Web sites of PLoS Medicine at http://www.plosmedicine.org/, Annals of Internal Medicine at http://www.annals.org/, and Epidemiology at http://www.epidem.com/). Information on the STROBE Initiative is available at www.strobe-statement.org.

For peer review only
